# Ginkgolic acid inhibits orthopneumo- and metapneumo- virus infectivity

**DOI:** 10.1038/s41598-024-58032-8

**Published:** 2024-04-08

**Authors:** Maria I. Luck, Erick J. Subillaga, Ronen Borenstein, Yosef Sabo

**Affiliations:** 1 Aaron Diamond AIDS Research Center, Columbia University Vagelos College of Physicians and Surgeons, New York, NY 10032 USA; 2 Division of Infectious Diseases, Department of Medicine, Columbia University Vagelos College of Physicians and Surgeons, New York, NY 10032 USA; 3 The Program for Experimental and Theoretical Modeling Division of Hepatology, Department of Medicine Stritch School of Medicine, Loyola University Chicago, Maywood, IL 60153 USA

**Keywords:** Virology, Antivirals

## Abstract

The human respiratory syncytial virus (hRSV) and the human metapneumovirus (hMPV) are important human respiratory pathogens from the *Pneumoviridae* family. Both are responsible for severe respiratory tract infections in infants, young children, elderly individuals, adults with chronic medical conditions, and immunocompromised patients. Despite their large impact on human health, vaccines for hRSV were only recently introduced, and only limited treatment options exist. Here we show that Ginkgolic acid (GA), a natural compound from the extract of *Ginkgo biloba*, with known antiviral properties for several viruses, efficiently inhibits these viruses’ infectivity and spread in cultures in a dose-dependent manner. We demonstrate that the drug specifically affects the entry step during the early stages on the viruses’ life cycle with no effect on post-entry and late stage events, including viral gene transcription, genome replication, assembly and particles release. We provide evidence that GA acts as an efficient antiviral for members of the *Pneumoviridae* family and has the potential to be used to treat acute infections.

## Introduction

*Pneumoviridae* family viruses are enveloped, single-stranded, negative-sense RNA viruses belonging to two genera—orthopneumovirus and metapneumovirus^1^*.* The human respiratory syncytial virus (hRSV) and the human metapneumovirus (hMPV), members of the two genera, are important human respiratory pathogens. They are the most common cause of severe upper and lower respiratory tract infections in infants and young children, and are also a major pathogen in elderly individuals, adults with chronic medical conditions, and immunocompromised patients. They are the leading cause of hospitalization for infants and are the most common cause of bronchiolitis and pneumonia in children younger than 1 year of age in the United States^2–18^. Until recently, there were only two therapies approved for hRSV, with limited efficiency—an antiviral drug (Ribavirin;^19,20^), which is used in severe high-risk cases, and a humanized monoclonal antibody that targets the viral fusion protein (Palivizumab;^21^). Recently, two hRSV vaccines were approved by the FDA for adults 60 years and older (Arexvy by GSK and Abrysvo by Pfizer;^22,23^) and pregnant women (Abrysvo by Pfizer;^24^), as well as a preventive hRSV humanized monoclonal antibody treatment for infants (Nirsevimab;^25,26^). For hMPV, no licensed vaccines or drugs exist. Taken together, the status quo indicates that finding new therapeutics for these viruses is of high importance.

Ginkgo biloba extracts from the leaves and fruits of the Ginkgo biloba contain a wide variety of chemical compounds^27^, including Ginkgolic acid (GA)^28^. GA, and in particular GA C15:1, which is the major compound found in these extracts^28^, has been widely researched as a therapeutic option for various human diseases^29–33^ including viral infections^34–41^. In vitro studies of GA treatment revealed broad antiviral activity. Infectivity of herpes simplex virus type 1, human cytomegalovirus, and the non-enveloped adenovirus type 5 was shown to be severely inhibited upon GA treatment^36^. Similar observation of reduction in infectivity was also reported for the coronavirus Strain 229E after GA treatment^34^. For the arboviruses Chikungunya, Mayaro, Una, and Zika viruses, GA was reported to possess several antiviral activities including direct virucidal activity, inhibition of viral proteins expression, and reducing viral titers^37^. In addition, using a virus-free model of virus-cell fusion by expressing viral fusion proteins on target cells and monitoring cell-to-cell fusion events, GA treatment was demonstrated to prevent the fusion of cells expressing the fusion proteins of Zika virus, HIV-1, Ebola virus, influenza A virus, Semliki Forest virus, Venezuelan equine encephalitis virus, vesicular stomatitis virus, and Epstein Barr virus^36^. Recently, GA treatment was also reported to block the interaction between the viral glycoprotein of the Severe acute respiratory syndrome coronavirus 2 (SARS‑CoV‑2)—the spike protein—and its host cell receptor ACE2, thus preventing viral entry^40^. GA was also shown to inhibit the proteolytic activities of the HIV-1 protease^39^ and the main protease of SARS-CoV-2 (the 3CLpro)^38,41^. Lastly, in vivo studies of GA treatment demonstrated the drug ability to hamper HSV-1 skin infection in BALB/c mice, thus preventing zosteriform spread^35^. Altogether, these studies demonstrated the therapeutic antiviral potential of GA. Yet, for members of the *Pneumoviridae* family no work describing GA effect on viral infectivity has been reported.

Here we report the antiviral activity of GA against two members of the *Pneumoviridae* family—hRSV and hMPV. We show that GA specifically and directly affects the entry step during the early events of the viruses’ life cycle, and that this inhibition is observed at drug concentrations below the cytotoxic threshold. Furthermore, we demonstrate that GA treatment does not affect viral post-entry events such as viral replication, viral gene transcription, viral assembly or particle release. Overall, the ability of GA to inhibit the viral entry of hRSV and hMPV, both important human pathogens, has a therapeutic potential that may be utilized in the clinic.

## Results

### Ginkgolic acid treatment efficiently inhibits hRSV spread in culture

**Figure 1:**
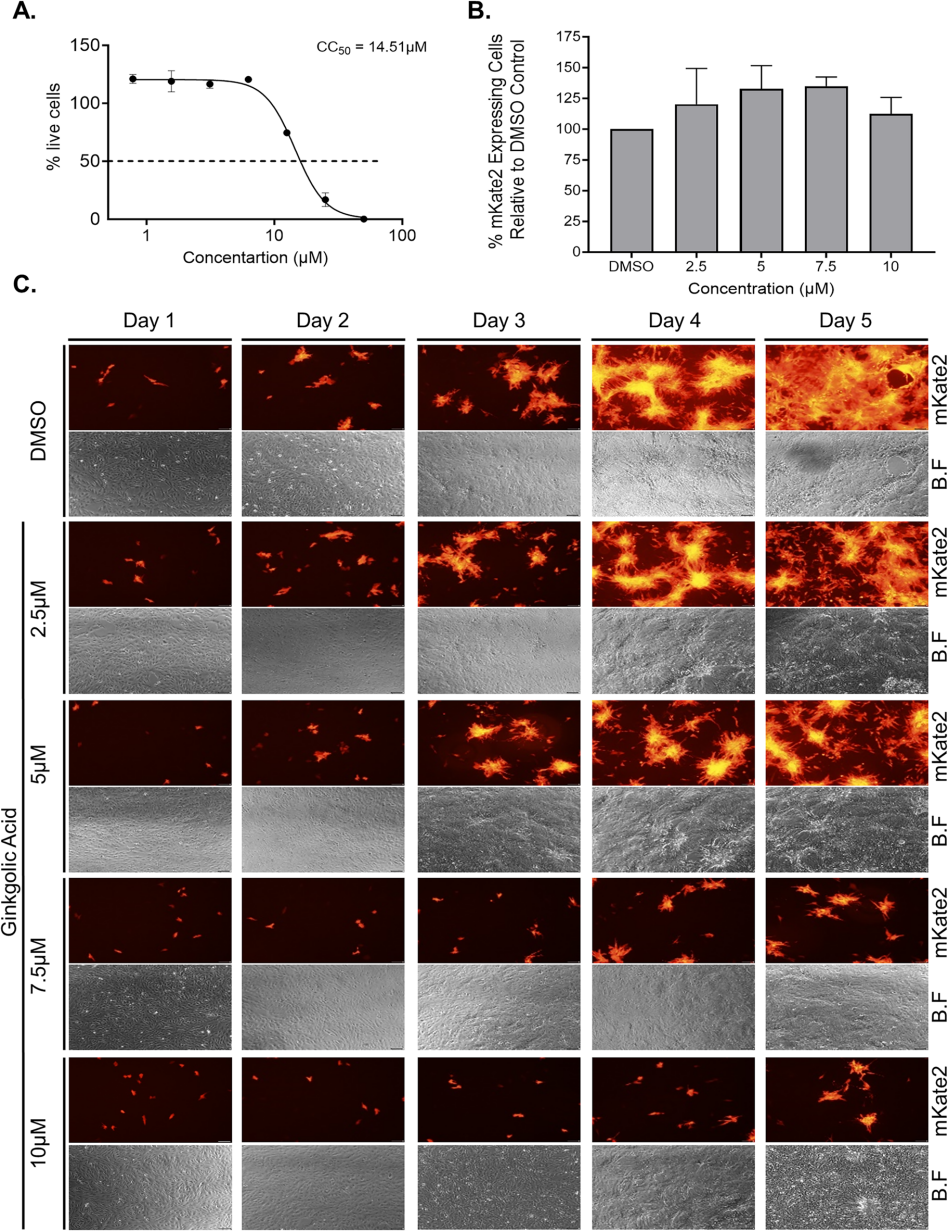
Respiratory syncytial virus spreading in culture in inhibited by Ginkgolic acid. (**A**) Cell cytotoxicity of Ginkgolic acid in Vero E6 cells. Cells were treated with Ginkgolic acid in a two-fold dilution series starting 50µM, for 5 days. CC_50_ value was assessed based on cell viability percentage compared to DMSO treated cells. (**B**) Flow cytometry 24 h post Ginkgolic acid treatment of Vero E6 cells infected with hRSV-mKate2. Cells were infected for 2 h with hRSV-mKate2 at a MOI of 0.05. Post infection the cells were washed 3 times with PBS and media containing DMSO of Ginkgolic acid at the indicated concentrations were added to the cultures for 24 h. The percentage of cells scoring red-positive relative to DMSO control (set as 100) are plotted. The data are mean ± SEM from three independent experiments (n = 3). (**C**) hRSV-mKate2 spread in Vero E6 cultures post Ginkgolic acid treatment. Cells were infected as in (B) and were daily monitored for the spread of hRSV-mKate2 using fluorescence microscopy to image the presence of the mKate2 fluorescent reporter. Shown are representative fields of view from a single experiment out of three independent experiments (n = 3). B.F, brightfield. Scale bar, 100 μm.

To test the inhibitory effect of GA on hRSV infection, we monitored hRSV spread in the presence or absence of the drug. To detect single infected cells during the spreading assay we used the hRSV-mKate2 reporter virus, which expresses the mKate2 fluorescent gene^42^. We used the African green monkey Vero E6 cells as they are deficient in interferon expression^43^ and thus susceptible and support the spread of hRSV for a prolonged period of time (up to 5 days) and display a distinct and easy to evaluate cytopathic effect (CPE). We first evaluated the potential cytotoxic effect of treating Vero E6 cells with GA for the period of time needed to monitor hRSV spreading. Vero E6 cells were treated with increasing concentration of GA for 5 days, after which cytotoxicity assay was performed and CC_50_ values were calculated (CC_50_ = 14.51 µM; Fig. 1A). We next tested the effect of GA treatment on the spread of hRSV. Vero E6 cells were infected with hRSV-mKate2 at a low multiplicity of infection (MOI) of 0.05 for 2 h. Post infection, the infection media was removed, the cells were extensively washed and media containing GA at varying concentrations or DMSO was added to the cultures. Every 24 h the cells were imaged using fluorescence microscopy to detect cells expressing the viral mKate2 reporter gene, which indicates a successfully infected cell. Prior to monitoring viral spread, we verified that in all tested conditions the first round of infection was unaffected by the GA treatment. Samples from each infected and DMSO or GA treated cultures were collected after 24 h and flow cytometry was performed to assess the number of infected cells (Fig. 1B). Indeed, no effect of GA treatment was observed after 24 h suggesting that viral entry was achieved prior to the drug treatment, and that the drug had no effect on the intracellular expression of the viral reporter gene (Fig. 1B and C). Monitoring viral spread using fluorescence microscopy at later time points revealed that for GA at concentrations above 2.5 µM, which is more than fivefold lower than the CC_50_ value of the drug, hRSV-mKate2 spreading was impaired compared to vehicle control (DMSO) throughout the entire course of the experiment (Fig. 1C). At day 4 and 5 post infection, large number of large multi-nucleate cells (syncytia), which are the hallmark phenotype of hRSV infection, could be detected in the infected and vehicle control treated cells (Fig. 1C and supplementary Fig. 1C and D). In contrast, cells infected and treated with GA at a concentration of 5 µM displayed markedly fewer small scattered clustered cells (Fig. 1C), and cells treated with 7.5 µM or 10 µM of GA showed only individual single mKate2-expressing cells (Fig. 1C).

Overall, the lower numbers of mKate2-expressing cells in GA-treated cultures at day 5 indicate that the drug prevents efficient viral spread in Vero E6 culture with no toxicity in the active range. The observation that the cells that were infected for 2 h and then treated with GA or DMSO for 24 h showed similar levels of the viral mKate2 reporter, which is transcribed by the viral transcription machinery from its genome, suggests the GA does not affect the virus post entry steps.

### Ginkgolic acid directly impairs the infectivity of the human respiratory syncytial virus

**Figure 2:**
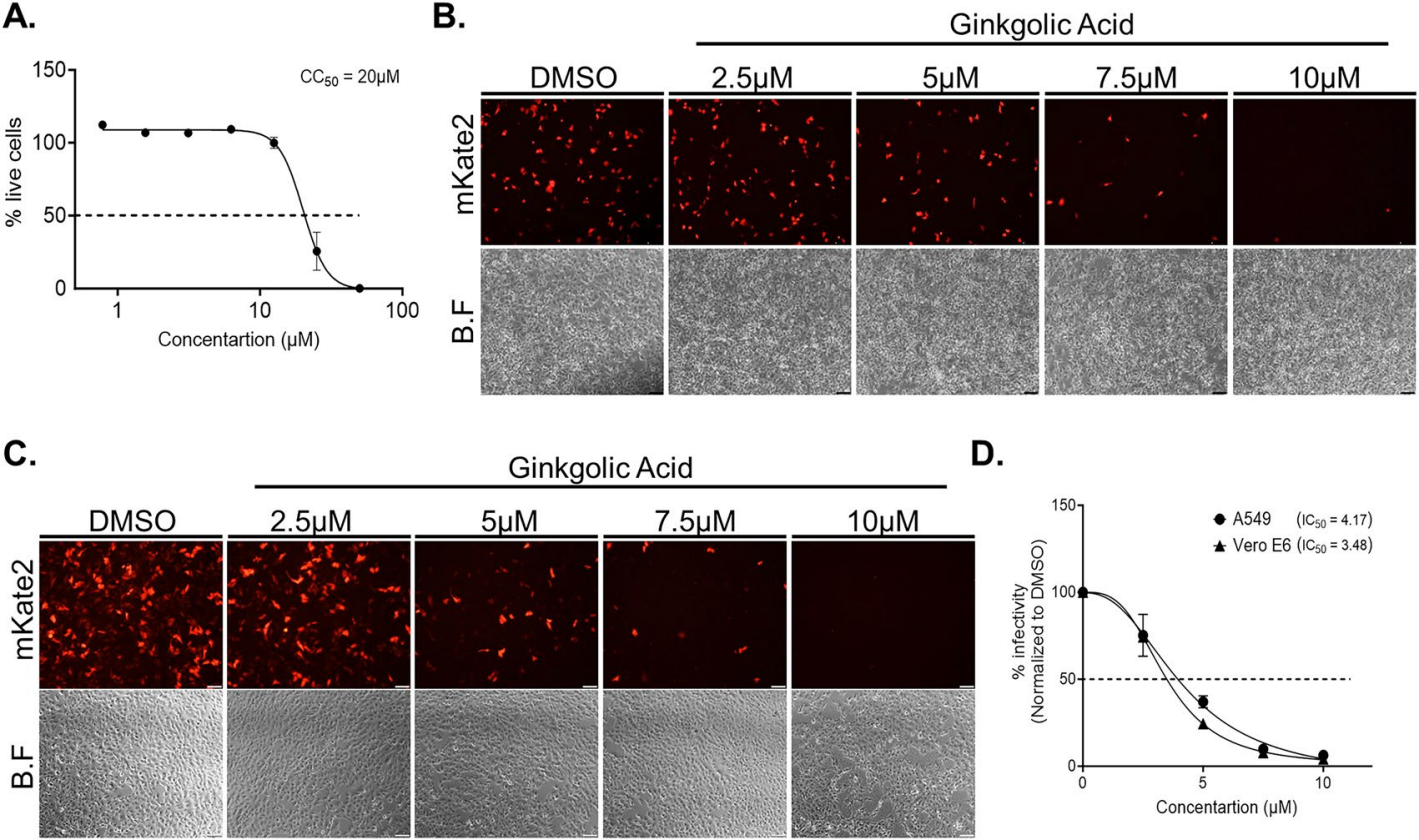
Infectivity of respiratory syncytial virus is inhibited post Ginkgolic acid treatment. (**A**) Cell cytotoxicity of Ginkgolic acid in A549 cells. Cells were treated with Ginkgolic acid in a two-fold dilution series starting 50 µM, for 96 h. CC_50_ value was assessed based on cell viability percentage compared to DMSO treated cells. The data are mean ± SEM from three independent experiments (n = 3). Fluorescence microscopy monitoring the expression of mKate2 fluorescent protein in A549 cells (**B**) or Vero E6 (**C**) infected for 24 h with hRSV-mKate2 viruses that were pre-treated for 1 h with DMSO or the indicated Ginkgolic acid concentration. Shown are representative fields of view from a single experiment out of three independent experiments (n = 3). B.F, brightfield. Scale bar, 100 μm. (**D**) Flow cytometry of cells from (**B**) and (**C**) was used to determine the number of mKate2 expressing cells in each cultures and calculate the IC_50_ values of Ginkgolic acid. The data are mean ± SEM from three independent experiments (n = 3).

We next sought to test if GA has a direct effect on the infectivity of hRSV, specifically during the entry step of the virus life cycle. Since hRSV is a human pathogen that infect the lower respiratory tract, we switched our experimental system to use the human lung epithelium A549 cell-line, which is more suitable for the study of hRSV^44^. As above, we initially determined CC_50_ values for GA treatment on A549 cells by adding GA at increasing concentrations or vehicle DMSO for 24 h and performing cytotoxicity assays (Fig. 2A). Next, hRSV-mKate2 viruses at a MOI of 0.2 were incubated in serum-free medium for 1 h at 37 °C with either GA at various concentrations or DMSO. Post incubation, serum was added to each of the treated samples to a final concentration of 1%, and the pre-treated viruses were used to infect A549 cells. After 24 h, the cultures were examined using a fluorescent microscope to detect infected mKate2-expressing cells (Fig. 2B). Reduction in the infectivity of hRSV-mKate2 could be detected after treatment with GA at a concentration of 5 µM, and no infected cell could be detected at a concentration of 10 µM (Fig. 2B). Flow cytometry to quantify the number of infected cells and calculate the IC_50_ value of the drug (IC_50_ = 4.17 µM; Fig. 2C) further confirmed the microscopy observations. Similar results were obtained when the same experiments were done using Vero E6 cells infected with hRSV-mKate2 viruses at a MOI of 0.8 (IC_50_ = 3.48 µM; Fig. 2C–D) indicating that GA is a virucidal agent, and its treatment directly impairs viral infectivity in both cell lines tested.

### Respiratory syncytial virus replication and virion production are not affected by Ginkgolic acid

**Figure 3:**
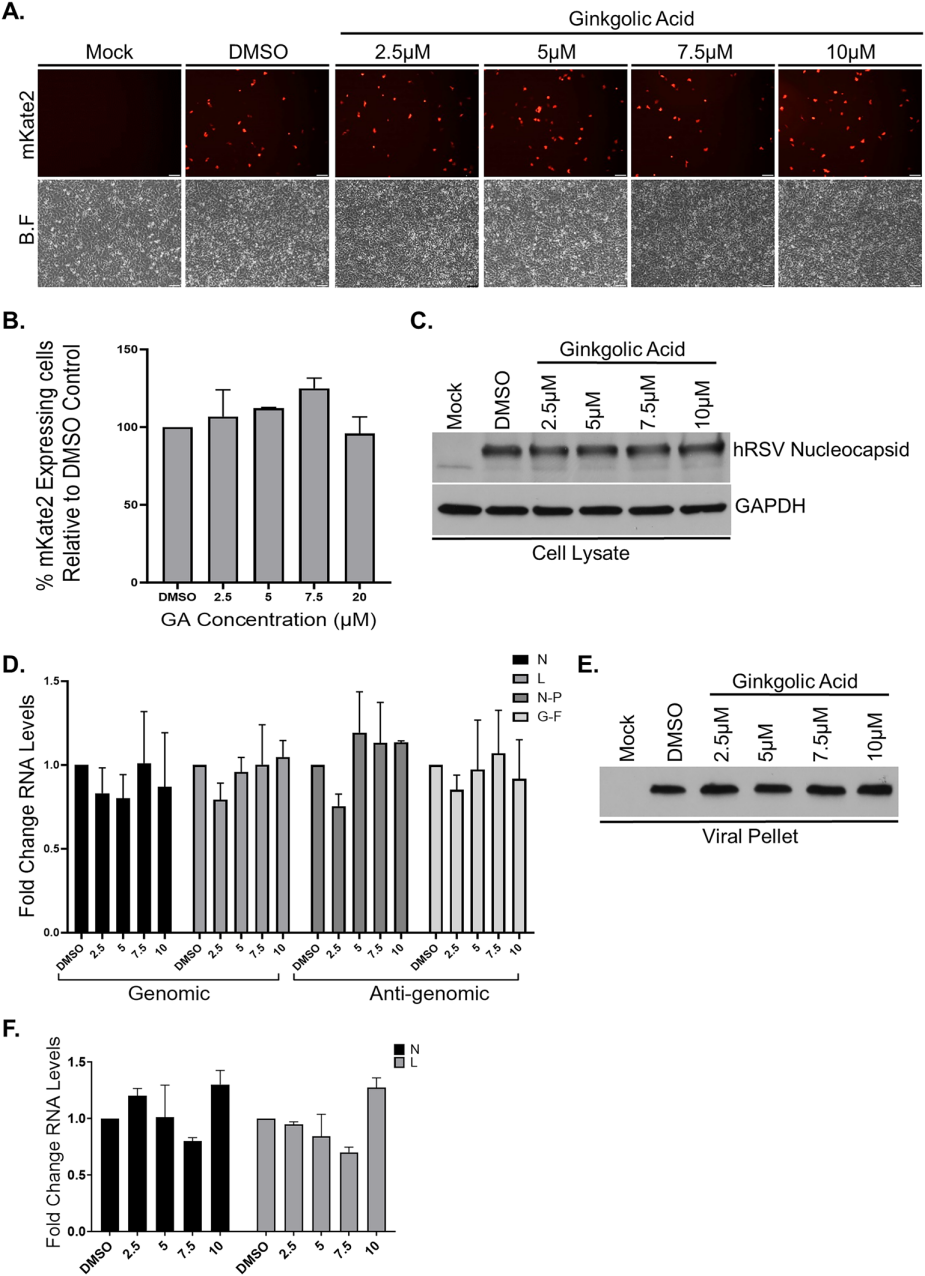
Respiratory syncytial virus assembly and release are not affected by Ginkgolic acid treatment. A549 cells were infected with hRSV-mKate2 for 2 h, after which the cells were washed with PBS and media containing DMSO or GA at various concentration were added to the cultures for 24 h. (**A**) Fluorescence microscopy to detect the expression of mKate2 fluorescent protein in infected and treated cells. Shown are representative fields of view from a single experiment out of three independent experiments (n = 3). B.F, brightfield. Scale bar, 100 μm. (**B**) Flow cytometry of cells from (A) to quantify the number of mKate2 expressing cells. The percent cells scoring red-positive relative to DMSO control (set as 100) are plotted. The data are mean ± SEM from three independent experiments (n = 3). (**C**) hRSV Nucleocapsid expression levels from infected and treated cell lysates were assessed using western blot with specific anti-hRSV Nucleocapsid antibody. Shown are representative western blots from three independent experiments (n = 3). (**D**) viral genomic and anti-genomic RNA levels form infected and treated cell lysates were assessed using quantitative reverse transcription polymerase chain reaction (qRT-PCR). Two different primer sets were used for each RNA species—primers for N and L ORFs were used to measure genomic RNA levels, primers for the regions spanning between N and P ORFs or F and G ORFs were used to measure the levels of the anti-genomic RNA. The fold change of viral genomic or anti-genomic RNA levels normalized to microtubules mRNA levels in GA treated compared to DMSO treated are plotted. The data are mean ± SEM from three independent experiments (n = 3). (**E**) Virions from culture supernatants of (C) were isolated by pelleting through 25% sucrose cushions and hRSV Nucleocapsid expression levels were assessed as in (C). Shown is a representative western blot from three independent experiments (n = 3). (**F**) Virions from culture supernatants of (D) were isolated by pelleting through 25% sucrose cushions and qRT-PCR using two primer sets (amplifying in N and L ORFs) was used to measure the levels of genomic RNA in each sample. The fold change of genomic RNA levels in GA treated compared to DMSO treated cultures are plotted. The data are mean ± SEM from three independent experiments (n = 3).

Our data suggest that GA treatment inhibit viral entry into its target cells (Fig. 2) without affecting viral replication and gene transcription post entry (Figs. 1 and 2). It is nevertheless possible that GA treatment interferes with other aspects of the viral late events of its life cycle and prevents proper viral assembly and release. We next evaluated GA involvement in the production and release of hRSV viral particles from infected cells. A549 cells were infected for 2 h with hRSV-mKate2 at a low MOI of 0.05 to duplicate our spreading assay infection conditions. Post infection, the cells were intensively washed and media containing DMSO or various concentrations of GA were added for 24 h. Fluorescent microscopy to detect the expression of the mKate2 reporter gene in the infected cultures at 24 h post infection showed equivalent viral infectivity across all treatments (Fig. 3A). Flow cytometry of the cultures further confirmed this observation (Fig. 3B). Analysis of cell lysates using western blot (WB) probed with anti-hRSV Nucleocapsid antibody, showed comparable amount of viral Nucleocapsid proteins in DMSO or GA treated cells (Fig. 3C and Supplementary Fig. 3B and D). Quantitative reverse transcription polymerase chain reaction (qRT-PCR) of the cell lysates using primers sets to detect viral genome or anti-genome levels demonstrated equivalent amounts of genomic RNA between all tested treatments as well as similar levels of anti-genomic RNA in all treated cultures, suggesting that viral replication was unaffected by GA treatment (Fig. 3D). Examination of the virion particles pelleted through a 25% sucrose cushion from the culture medium by WB and by qRT-PCR to measure viral genome levels, demonstrated comparable yields of virus proteins and genomic RNA with and without drug (Fig. 3E-F and Supplementary Fig. 3C and D), indicating that treatment with GA had no effect on viral assembly and virion release.

To rule out the possibility that GA is a slow acting drug, which might require more than 24 h to show any effect on viral replication, assembly and release, we treated A549 cells for 24 h with DSMO or GA at various concentrations. Post treatment, the cells were infected for 2 h with hRSV-mKate2 at MOI of 0.05 in media supplemented with 1% FBS. The cultures were then extensively washed and media containing DMSO or GA were added for additional 24 h. As before, Flow cytometry of the cultures to measure the expression of the viral reporter mKate2 gene, and qRT-PCR to measure viral genome or anti-genome in the cells as well a qRT-PCR to measure the levels of viral genomic RNA in pelleted viruses showed no difference in DMSO treated cultures compared to GA treated ones (Supplementary Fig. 3E–G).

Altogether, these experiments further strengthen our initial observation that GA treatment does not affect post-entry events such as viral intracellular replication and gene expression. In addition, our data showed that hRSV viral late events including assembly and release are also not affected post GA treatment.

### Ginkgolic acid directly inhibit the infectivity of the human metapneumovirus without affecting post-entry viral replication, assembly and release

**Figure 4:**
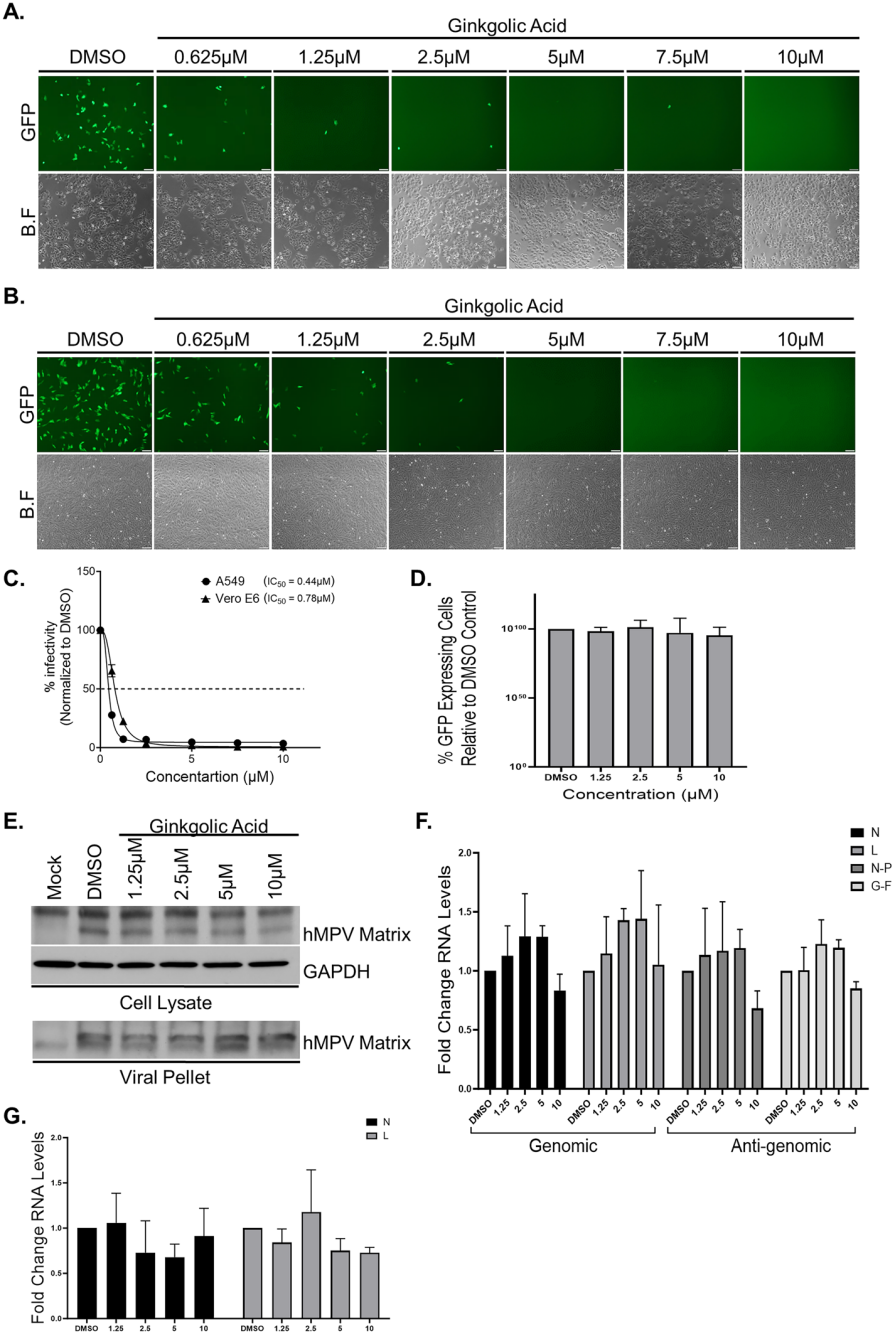
Ginkgolic acid inhibits the infectivity of human metapnuemovirus without affecting viral assembly and release. (**A**–**B**) Fluorescence microscopy monitoring the expression of GFP fluorescent protein in A549 cells (**A**) or Vero E6 cells (**B**) infected for 24 h with hMPV-GFP viruses that were pre-treated for 1 h with DMSO or the indicated Ginkgolic acid concentration. Shown are representative fields of view from a single experiment out of three independent experiments (n = 3). B.F, brightfield. Scale bar, 100 μm. (**C**) Flow cytometry (FACS) of cells from (A) and (B) was used to determine the number of GFP expressing cells in each cultures and calculate the IC_50_ values of Ginkgolic acid. The data are mean ± SEM from three independent experiments (n = 3). (**D**–**G**) A549 cells were infected with hMPV-GFP for 2 h, after which the cells were washed with PBS, and media containing DMSO or GA at various concentration were added to the cultures. After 24 h the culture supernatants were collected, and the cells were harvested and lysed. Virions in the supernatants were isolated by pelleting through 25% sucrose cushions. (**D**) FACS analysis to quantify the number of GFP expressing cells. The percent live cells scoring green-positive relative to DMSO control (set as 100) are plotted. The data are mean ± SEM from three independent experiments (n = 3). (**E**) hMPV Matrix protein in the cells (cell lysate) and in released virions (viral pellet) were detected by hMPV Matrix-specific antibody. Shown are representative western blots from three independent experiments (n = 3). (**F**) viral genomic and anti-genomic RNA levels form cell lysates were assessed using quantitative reverse transcription polymerase chain reaction (qRT-PCR). Two different primer sets were used for each RNA species—primers for N and L ORFs were used to measure genomic RNA levels, primers for the regions spanning between N and P ORFs or F and M2 ORFs were used to measure the levels of the anti-genomic RNA. The fold change of viral genomic or anti-genomic RNA levels normalized to microtubules mRNA levels in GA treated compared to DMSO treated are plotted. The data are mean ± SEM from three independent experiments (n = 3). (G) qRT-PCR of virion pellets using two primer sets (amplifying in N and L ORFs) were used to measure the levels of genomic RNA in each sample. The fold change of genomic RNA levels n in GA treated compared to DMSO treated are plotted. The data are mean ± SEM from three independent experiments (n = 3).

We next tested whether the inhibition observed for hRSV infectivity after GA treatment can be extended to other viruses from the *Pneumoviridae* family. hMPV, another member of this family is also a major human respiratory pathogen that although mainly responsible for upper respiratory infection displays similar clinical manifestations to hRSV infection^4–9,11–15,18^. hMPV-GFP virus^45^ expressing a green fluorescent protein (GFP) from its genome, was incubated in serum free medium for 1 h at 37 °C with either DMSO or GA. Post the incubation period, serum and trypsin at a final concentration of 1%, and 0.25 mg/ml, respectively, were added to each of the treated samples. The treated viruses were then used to infect A549 cells at MOI of 0.1 for 24 h, after which the infected cultures were examined using a fluorescence microscope to detect infected, GFP expressing cells (Fig. 4A). In addition, flow cytometry was used to quantify the number of live GFP-positive cells and determine the drug IC_50_ value (IC_50_ = 0.44 µM; Fig. 4C). Strikingly, the infectivity of hMPV was inhibited by a markedly lower GA concentration treatment (Fig. 4A and C) compared to the concentration needed to inhibit hRSV (Fig. 2). We next tested if the observed lower concentration of GA needed to inhibit hMPV compared to hRSV in A549 cells was also observed with Vero E6 cells. Infecting Vero E6 cells with DMSO or GA pre-treated hMPV-GFP showed similar results with an IC_50_ value of 0.78 µM (Fig. 4B and C).

As we have done above for hRSV, we evaluated GA involvement in the production and release of hMPV viral particles from infected cells. A549 cells were infected for 2 h with hMPV-GFP at a low MOI of 0.05, and the cells were intensively washed and DMSO or GA containing media was added for 24 h. Post treatment, flow cytometry used to evaluate the number of GFP positive cells, demonstrated equal infectivity across all treatments (Fig. 4D). WB analysis of cell lysates and virion particles pelleted through a 25% sucrose cushion from the cultures medium, and probed with anti-hMPV Matrix antibody showed comparable amount of the viral Matrix protein (Fig. 4E and Supplementary Fig. 4C and D). qRT-PCR to measure the levels of viral genome or anti-genome RNAs in the cells and genomic RNA levels in viral particles pelleted through a 25% sucrose cushion, demonstrated equivalent levels for each of the specific RNAs in all tested conditions (Fig. 4F and G). Similar results were obtained when cultures were treated with GA 24 h prior to hMPV-GFP infection, after which they were infected for 2 h without the drug and then were incubated for additional 24 h in the presence of the drug (Supplementary Fig. 4E–G).

Overall, these observations suggest that while GA treatment is a strong inhibitor of viral infectivity, it does not affect viral intracellular replication, virus assembly, or virion release for members of the *Pneumoviridae.*

## Discussion

We have identified GA as a potent inhibitor of infectivity and spread in culture for hRSV and hMPV, both members of the *Pneumoviridae* family. We showed that the inhibition of infectivity occurs during the entry step in the early stage of the viruses’ life cycle with no effect on late events such as viral replication, gene expression, and viral assembly and release.

GA was suggested to inhibit protein SUMOylation in vitro and in vivo by binding E1 and preventing the formation of the E1-SUMO intermediate^46^. While we cannot exclude that treatment with GA had some undetected influence on the tested cells, our data showed that viral post-entry events were still able to take place successfully in the presence of the drug. The equivalent intracellular expression levels of the mKate2 or GFP reporters and the viral Nucleocapsid or Matrix genes (Figs. 3 and 4), and the similar cellular RNA levels of viral genome or viral anti-genome in the DMSO or GA treated cells, suggest that the viruses transcription and replication machinery as well as the cellular translation machinery were unaffected by GA treatment. Further supporting this is the observation that viral particles can be released from all treated cultures and that these released particles share similar properties such as their ability to be pelleted through a sucrose cushion, their similar levels of Nucleocapsid or Matrix proteins and the equivalent levels of packaged genomic RNAs (Figs. 3 and 4).

For several viruses GA treatment was reported to have a broad anti-viral activity not limited to the inhibition of viral infectivity during the entry step of the viral life cycle. GA was shown to inhibit viral gene expression^37^, reducing the amount of released viral particles from infected cells^37^, and inhibit the function of key viral enzymes^38,39,41^. Nevertheless, our data suggest that GA treatment does not affect hRSV or hMPV post-entry events. Our data clearly show that adding GA post infection to the cultures, even at a very high dosage, had no influence on viral gene expression, viral genome replication or viral particles assembly and release when compared to control treated cultures. (Figs. 1, 3 and 4), thus, providing evidence that GA has no role in post-entry late-stages for viruses from the *Pneumoviridae* family and only affects the entry step of these viruses’ life cycle. The lack of GA effect on viral particle release suggests that the spreading reduction seen in Fig. 1 post infection and following GA treatment, is due to inhibition of viral entry as viruses can be made and release but due to their exposure to GA, which is present in the medium, cannot infect new cells and thus spread. As such, we cannot test the infectivity of the released viral particles and cannot exclude the possibility that GA treatment may contribute to an increase in the release of defective particles that are non-infectious. Since GA mechanism of actions is not yet fully determined, further studies will be required to assess this option.

Our data demonstrating that GA only affect viral entry is in line with observations reported for several viral pathogens^34,36,40^, which also did not report any post-entry anti-viral activity. Yet, for some other viruses GA was shown to have post-entry antiviral activity^37–39,41^. This suggests that GA has several mechanism of actions, which affect in a different way different viruses—all yet to be discovered.

The difference in GA potency on hRSV compared to hMPV infectivity (Figs. 2 and 4) hints that although GA seems to be a pan-acting entry inhibitor of many viruses^34,37,40,47^, some specificity to viral glycoproteins exists. This raises the possibility that modifications of GA might improve its antiviral efficacy making it a better pan-acting antiviral at lower concentration.

In conclusion, the ability of GA to inhibit the infectivity of two members from the *Pneumoviridae* family, hRSV and hMPV, hints that the drug may have a broader range of action and can be used to treat other members of this family as well. Further modification aiming to improve the molecule potency in inhibiting viral entry while reducing its toxicity are under way.

## Methods

### Cells

Vero E6, HEK293T and A549 cells were purchased from ATCC (Cat# CRL-1586, Cat# CRL-11268 and Cat# CCL-185; respectively) and maintained in Dulbecco’s Modified Eagle Medium containing GlutaMAX (DMEM; GIBCO, Cat# 10566-016) and supplemented with 10% fetal bovine serum (FBS), 100 U/ml penicillin and 100 μg/ml streptomycin (GIBCO, Cat# 15140-163).

### Viruses

hRSV-mKate2 reporter virus—a hRSV strain A2 carrying the fluorescent mKate2 gene, was generated using a bacterial artificial chromosome (BAC)-based reverse genetic system kindly provided by Dr. Martin Moore^42^ (The following reagents were obtained through BEI Resources, NIAID, NIH: BAC Plasmid pSynkRSV-I19F Containing antigenomic cDNA from RSV A2-Line19F, NR-36460; pA2-Lopt, pA2-Nopt, pA2-Popt and pA2-M2-1opt helper plasmids encoding for RSV A2 Large Polymerase, Nucleocapsid, phosphoprotein and Matrix 2–1; NR-36461, NR-36462, NR-36463 and NR-36464). hMPV-GFP reporter virus—a hMPV isolate NL/1/99 carrying the GFP gene, was rescued using a reverse genetic system described previously^45,48^. Briefly, to rescue each of the viruses, HEK293T cells in 100 mm plates were co-transfected with pT7 (a plasmid encoding for a T7 polymerase; 2 μg), a plasmid encoding for the viral antigenome (pSynkRSV-I19F or phMPV-GFP; 3 ug) and the 4 virus specific helper plasmids (3 ug for the polymerase [L] and nucleocapsid [N] plasmids and 2ug for the phosphoprotein and matrix 2–1 plasmids) using lipofectamine 3000 (ThermoFisher, Cat# L3000001) according to the manufacturer’s instruction. Two days post transfection the presence of the fluorescent reporter protein was monitored using fluorescence microscopy. Third of the transfected culture was resuspended in the culture supernatant and was co cultivated with 80% confluent Vero E6 cells in a 100mm plates. For hMPV-GFP rescue, the medium used for co-cultivation was adjusted to 3% FBS and was supplemented with 0.25 mg/ml trypsin. When cytopathic effect was observed, the cells were scraped from the plates, subjected to 3 fast freezing and thawing cycles, and cellular debris were pelleted by centrifugation at 1800×*g* for 5 min at 4 °C. Next, the cleared supernatants were layered on 25% sucrose cushions and centrifuged at 100,000×*g* for 2 h as described previously^45^. Viral pellets were resuspended in serum-free optimum media (GIBCO, Cat# 31985070). For each cell-line that was used for infection or spreading studies, viral titer and MOI calculation was determined by performing serial dilutions of the virus and infecting the appropriate cells for 24 h in a 12-well plate. Post infection, FACS analysis was used to evaluate the number of cells expressing the fluorescent reporter protein. All viral stocks were fully sequenced to verify no deletion or mutations occurred during the viral rescue process.

### Pelleting viral particles

Pelleting viral particles was performed as described previously^45^. Briefly, supernatants from DMSO or GA treated cultures were layered on 25%(w/v in 0.1M NaCl, 10mM Tris–Cl [pH 8], 1mM EDTA [pH 8]) sucrose cushions and centrifuged at 100,000×*g* for 2 h in Optima L-100 XP ultracentrifuge (Beckman Coulter). Viral pellets were resuspended in either TRIzol (ThermoFisher, Cat# 15596026) or 1 × Laemmli Sample Buffer (BIO-RAD; Cat# 161-0747) for RNA extraction or WB downstream usages, respectively.

### Virus whole genome sequencing

Viruses whole genome sequencing for viral stocks were performed as described by Quick et al.^49^. Viral RNAs were isolated from viral stocks using QIAamp Viral RNA Mini Kit (Qiagen, Cat# 52904) and reverse transcribed using LunaScript® RT SuperMix Kit (NEB, Cat# 3010) according to the manufactures instructions. For PCR amplification, primers designed to amplify 1000 base-pairs amplicons were tiled across the viral genome (https://primalscheme.com/,^49^), and PCR amplification was performed using Q5® Hot Start High-Fidelity 2X Master Mix (NEB, Cat# M0494). Consensus sequence generation was performed using the EPI2ME wf-alignment workflow (https://github.com/epi2me-labs/wf-alignment) with hRSV-mKate2 or hMPV-GFP reverse genetic genome sequences^42,45^.

### Viral genome and anti-genome quantification

To quantitate viral genome or anti-genome levels, RNA was extracted from cells or viral pellets using TRIzol (ThermoFisher, Cat# 15596026). For quantitative reverse transcription polymerase chain reaction (qRT-PCR) experiments, reverse transcription was performed using LunaScript® RT SuperMix Kit (NEB, Cat# 3010), and quantitative PCR was performed in an ABI 7500 Fast Real-Time PCR machine using FastStart Universal SYBR Green Master (Rox) (Millipore Sigma; Cat# 4913850001). All reagents were used according to the manufactures instructions. qPCR primers are listed in supplementary table 1.

### Drugs and antibodies

GA was purchased from Tocris (Cat# 6326) and dissolved in DMSO (Sigma, Cat# D8418) for a stock concentration of 100µM. Anti-GAPDH Mouse mAb (6C5), Rabbit IgG HRP Linked Whole Ab and Mouse IgG HRP Linked F(ab′)2 Fragment were purchased from Sigma (Cat# CB1001, NA934 and NA9310). Human respiratory syncytial virus (hRSV) strain A2 nucleoprotein antibody was purchased from Sino Biologicals (Cat# 40821-T46). Mouse anti-Human metapneumovirus matrix antibody (4811) was purchased from Cedarlane (Cat# MAB12287-100).

### GA treatment

For GA treatment, GA was diluted to the indicated working concentrations in DMEM supplemented with 1% FBS. To ensure equivalent amount of DMSO between all drugs dilutions, DMSO was added to each sample to a final volume of DMSO as in the highest drug working concentration. No drug control was DMSO.

### Viral infection

For infection prior to GA treatment, hRSV-mKate2 and hMPV-GFP were diluted in DMEM supplement with 1% FBS. For hMPV-GFP, trypsin was added to a final concentration of 0.25 mg/ml. Viruses were added to the cultures for 2 h at 37 °C. Post infection, the cells were washed three times with PBS (ThermoFisher, Cat# 14190250) and DMSO or GA diluted in DMEM supplement with 1% FBS were added to the cultures. For virus pre-incubation with GA experiments, viruses were diluted in serum-free DMEM and DMSO or GA were added at the indicated concentrations. The samples were incubated for 1 h at 37 °C, after which serum was added to a final concentration of 1%. Additionally, for hMPV-GFP, trypsin was added to a final concentration of 0.25 mg/ml. Samples were used to infect cells for 24 h r at 37 °C. Samples taken for FACS analysis were fixed with paraformaldehyde (Electron microscopy sciences, Cat# 15710) to a total of 4% and stored at 4 °C till analysis.

### Cytotoxicity assay

To evaluate the drugs cellular cytotoxicity, cells were seeded at a density of 2 × 10^4^ cells per well of a 96 wells plates. The following day, GA added to the cultures in a two-fold dilution series. All dilutions were done in DMEM supplemented with 1% FBS. At indicated times after addition of the drug, cytotoxicity was measured using Promega CellTiter-Glo® 2.0 Cell Viability Assay (Cat# G9243) reagent and CLARIOstar Plus (BMG LABTECH) plate reader. All experiments were done in triplicates. CC_50_ values were derived by fitting a nonlinear regression curve to the data in GraphPad Prism v.10.0.2 (Dotmatics).

### Quantification of viral infection and FACS

Quantification of viral infection was achieved by reading the number of live cells expressing the various fluorescent reporter proteins using BD LSR II Flow Cytometer with BD FACSDiva Version 8.0 software (BD Biosciences). Acquired data was analyzed using FlowJo version 10 (Tree Star).

### Microscopy

Monitoring mKate2 or GFP expressing cells was done at the indicated time points using Olympus ix73 microscope equipped with an Olympus UPlanFL N 10X/0.30na objective. Images were acquired using Olympus CellSens Standard 2.3 software.

### Western blots

Viruses from infected cells were purified by ultracentrifugation using a sucrose cushion as described previously ^45^ and lysed directly 1 × Laemmli Sample Buffer. Cells were lysed in RIPA (Sigma; Cat# R0278) for 30 min at 4 °C. The lysate was then cleared from cellular debris by centrifugation for 15 min at 16,110×*g* at 4 °C, and 4 × Laemmli Sample Buffer (BIO-RAD; Cat# 161-0747) supplemented with 20% β-Mercaptoethanol was added to the clear supernatant to a final concentration of 1×. Samples were then boiled for 5 min. Proteins were resolved by SDS-PAGE electrophoresis using a 4–20% Mini-PROTEAN TGX Precast Protein Gels (BIO-RAD; Cat# 4561094), transferred to a PVDF membrane (Millipore Sigma; Cat# IPFL00010) and probed by western blotting using the indicated antibodies. Primary antibodies were diluted 1:1000 and were incubated O.N at 4 °C. Secondary antibodies were diluted 1:10,000 and were incubated for 2 h at RT. Densitometric quantification was performed using ImageJ and the relative band intensity was normalized against GAPDH.

### Supplementary Information

Supplementary Information.

## Data Availability

All data generated and analyzed during this study are included in this published article and its supplementary information file.
