# Supplementary material for: Ginkgolic acid inhibits orthopneumo- and metapneumo- virus infectivity
Source: Sci Rep. 2024 Apr 8;14:8230. doi: 10.1038/s41598-024-58032-8 (PMC11001990; doi:10.1038/s41598-024-58032-8)
Supplement: Supplementary file 1 — Supplementary Information. [file 41598_2024_58032_MOESM1_ESM.pdf]

Supplementary materials

Ginkgolic acid inhibits Orthopneumo- and Metapneumo- virus infectivity

Maria I. Luck[1,2], Erick J. Subillaga[1,2], Ronen Borenstein[3], Yosef Sabo[1,2*]

[1] Aaron Diamond AIDS Research Center, Columbia University Vagelos College of Physicians and Surgeons, New York, NY, USA

[2] Division of Infectious Diseases, Department of Medicine, Columbia University Vagelos College of Physicians and Surgeons, New York, NY, USA

[3] The Program for Experimental and Theoretical Modeling Division of Hepatology, Department of Medicine Stritch School of Medicine, Loyola University Chicago Maywood, IL 60153, USA

* Address correspondence to Yosef Sabo (ys2581@cumc.columbia.edu, 212-304-6170, 701 W 168th St, 10th Floor, New York, NY 10032)

Supplementary figure 1

A.

Concentration uM	% normalized to no drug		
0.78125	113.4663	124.2909	125.3548
1.5625	104.1126	117.2457	135.6202
3.125	113.0236	112.8954	123.8478
6.25	121.6809	122.7858	117.2465
12.5	75.95374	73.61811	74.28109
25	25.52045	5.556264	19.41701
50	0.091527	0.101676	0.100904

B.

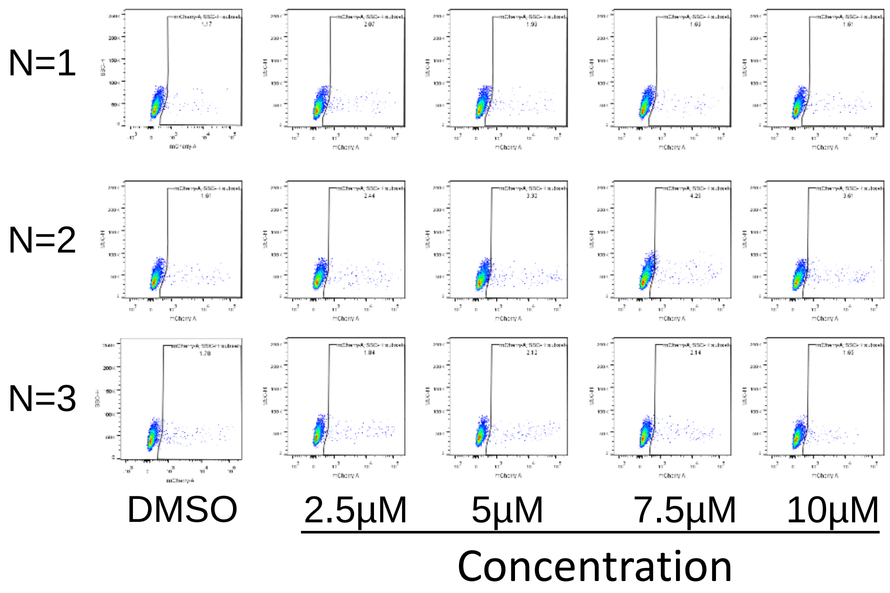

Concentration uM	Mean	SEM
DMSO	100	0
2.5	120.1857	29.14241
5	132.5797	18.99849
7.5	134.9336	7.45907
10	112.5134	13.23019

C.

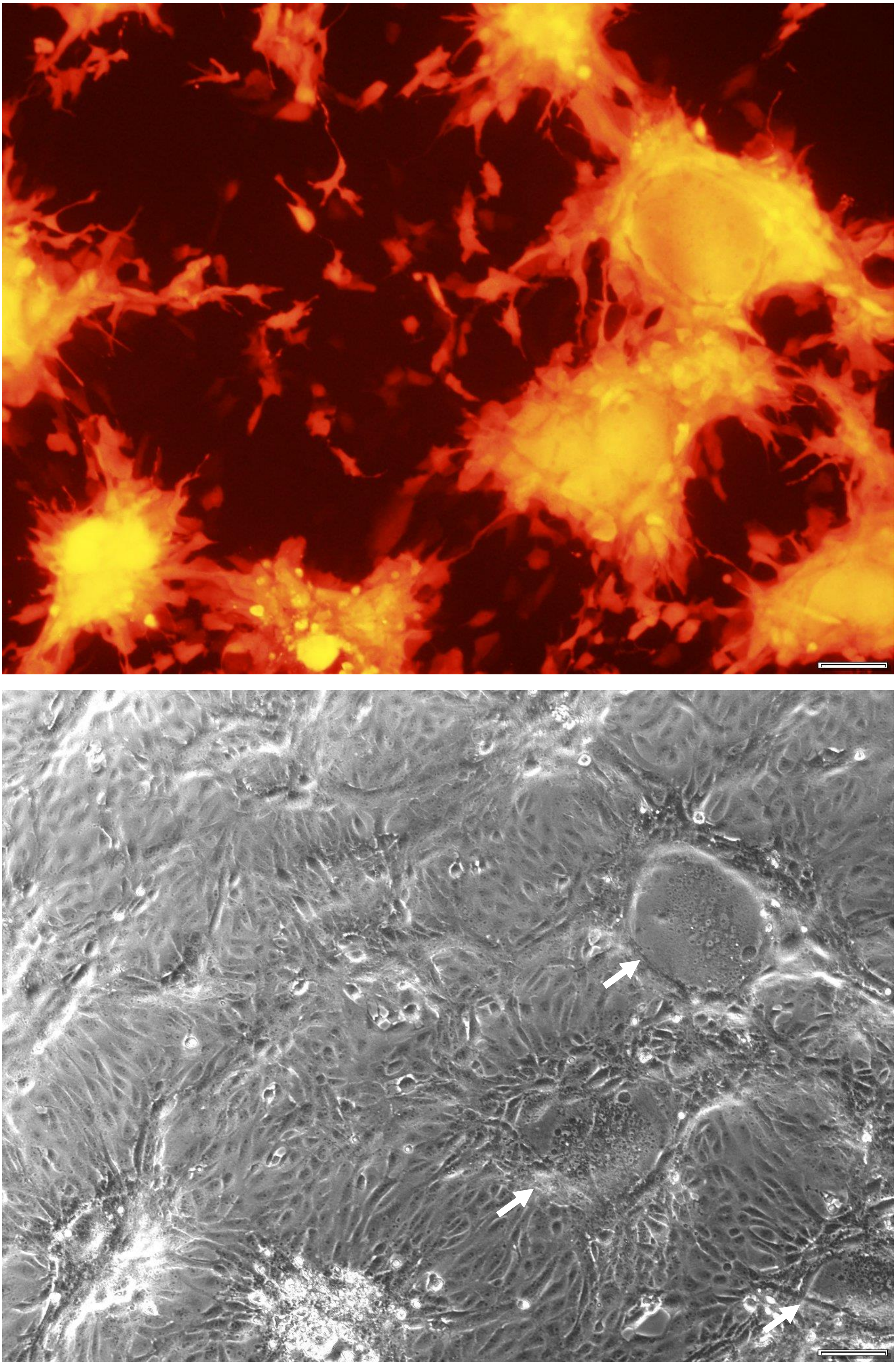

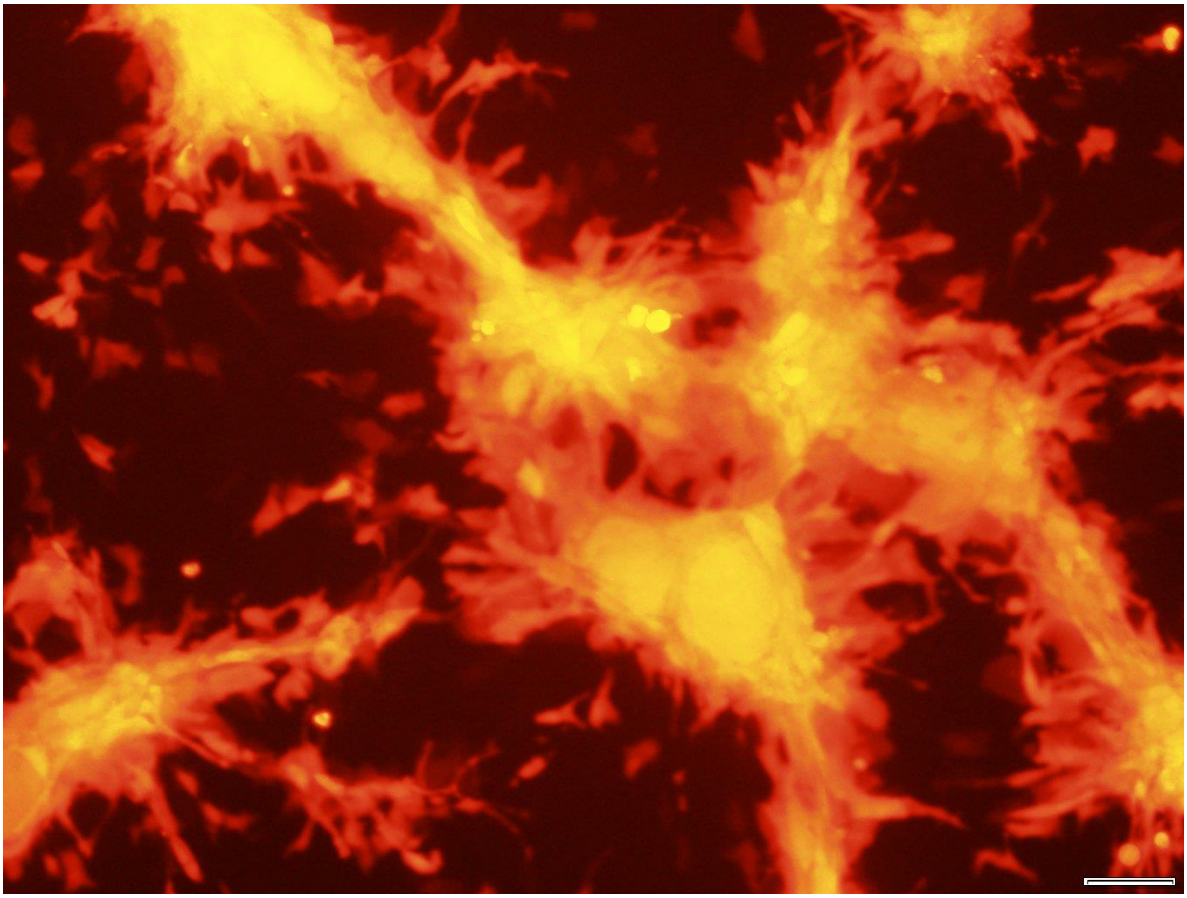

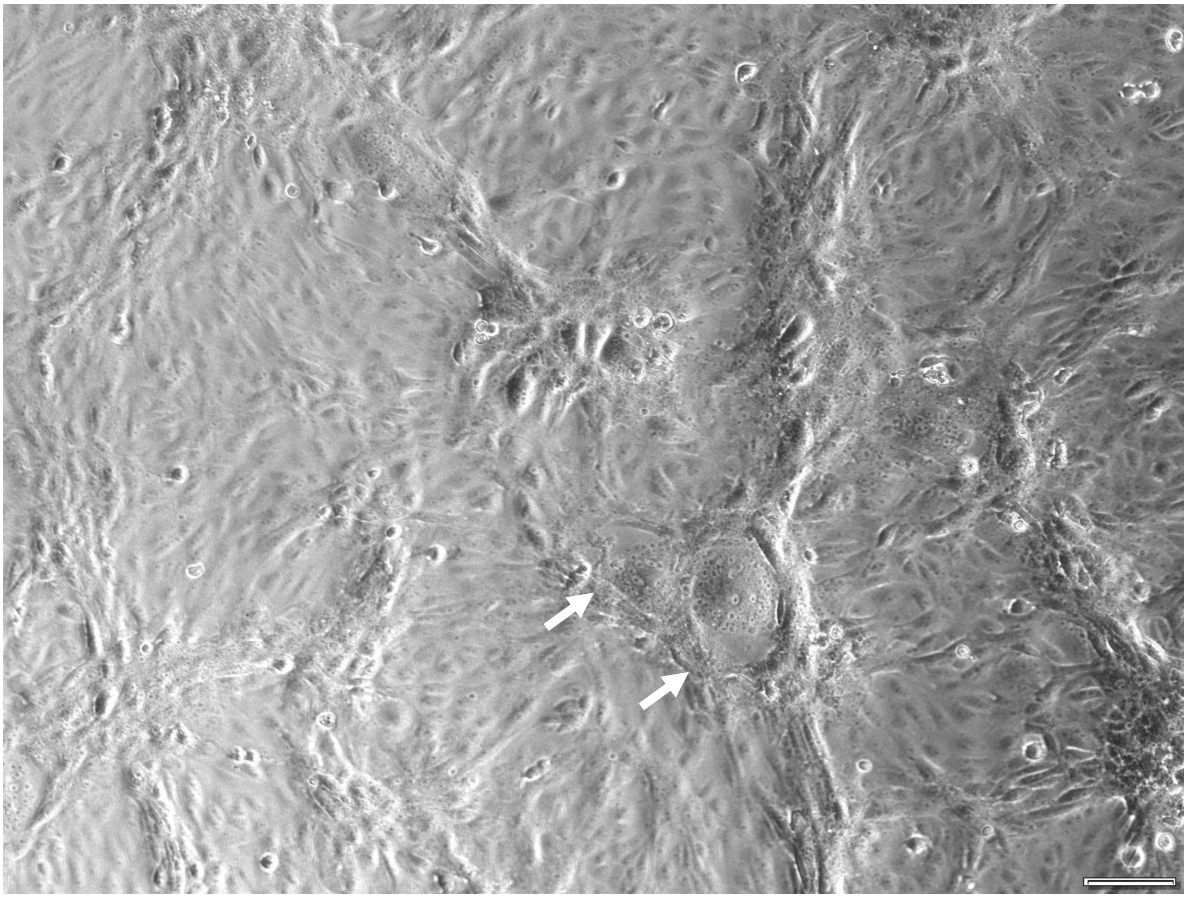

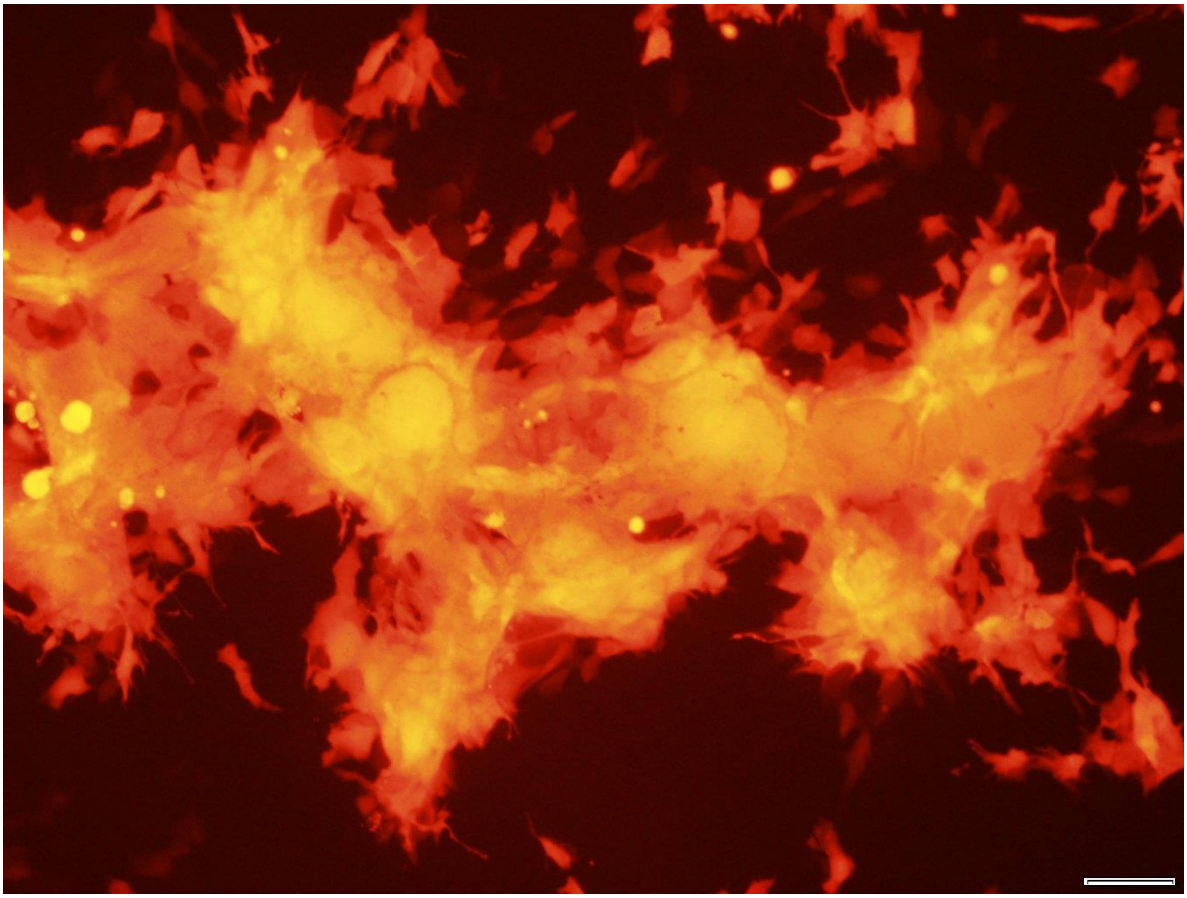

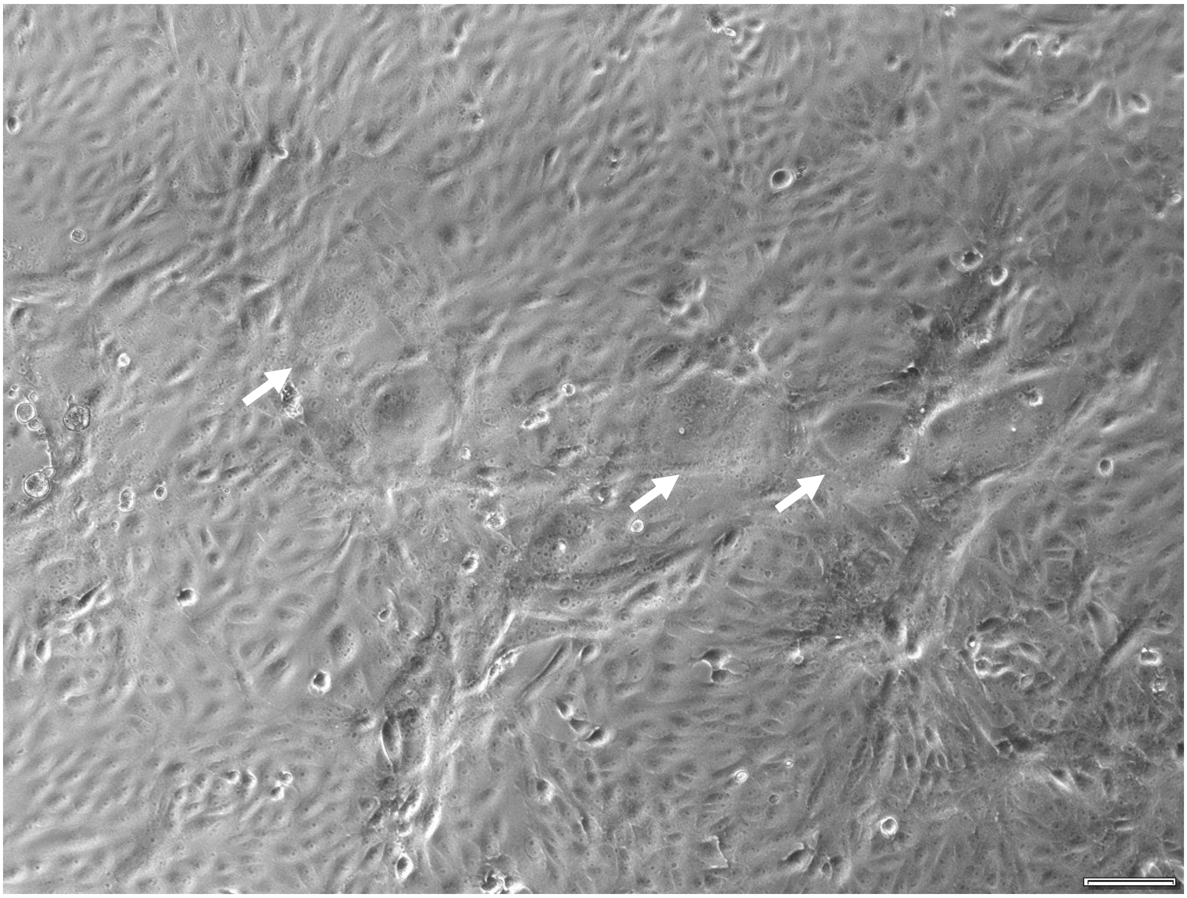

D.

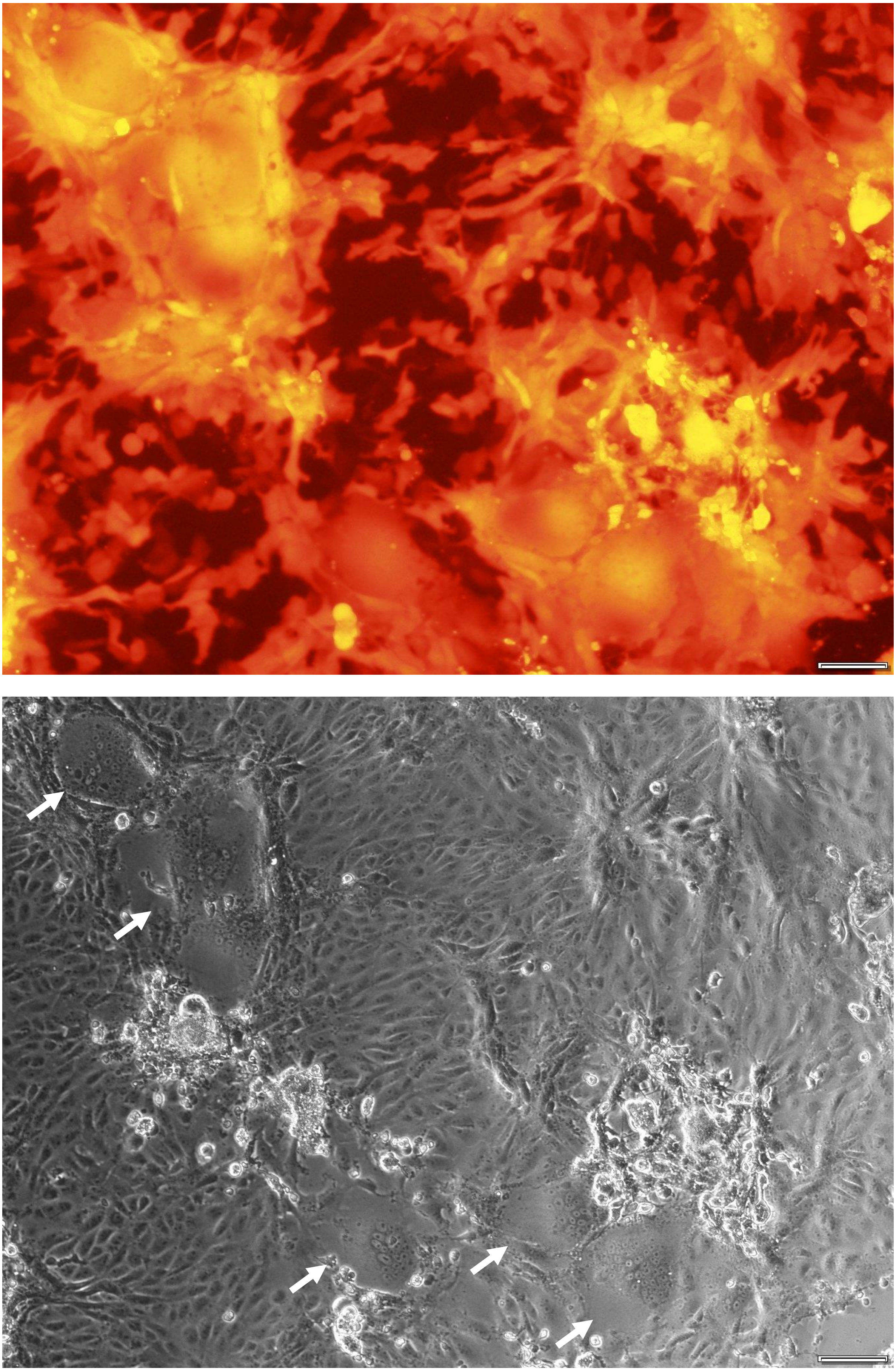

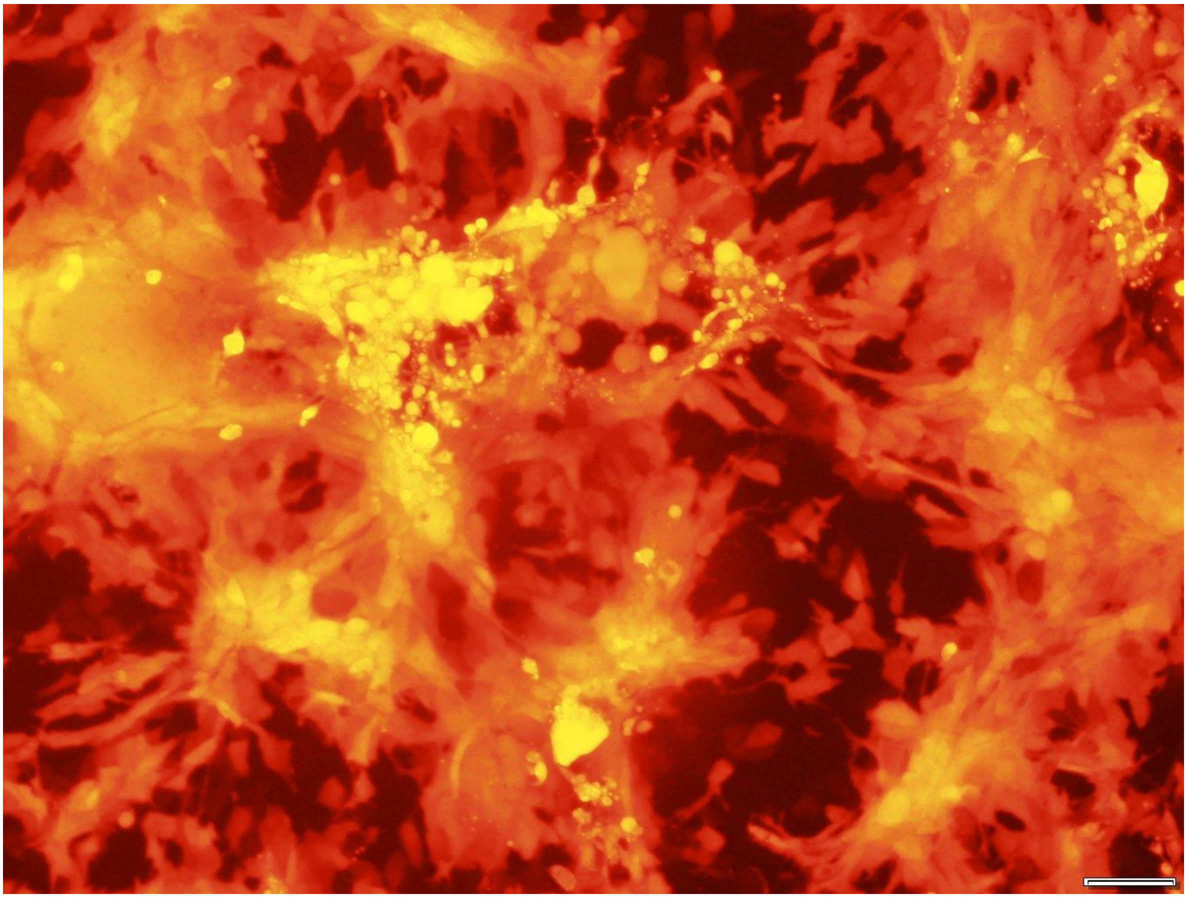

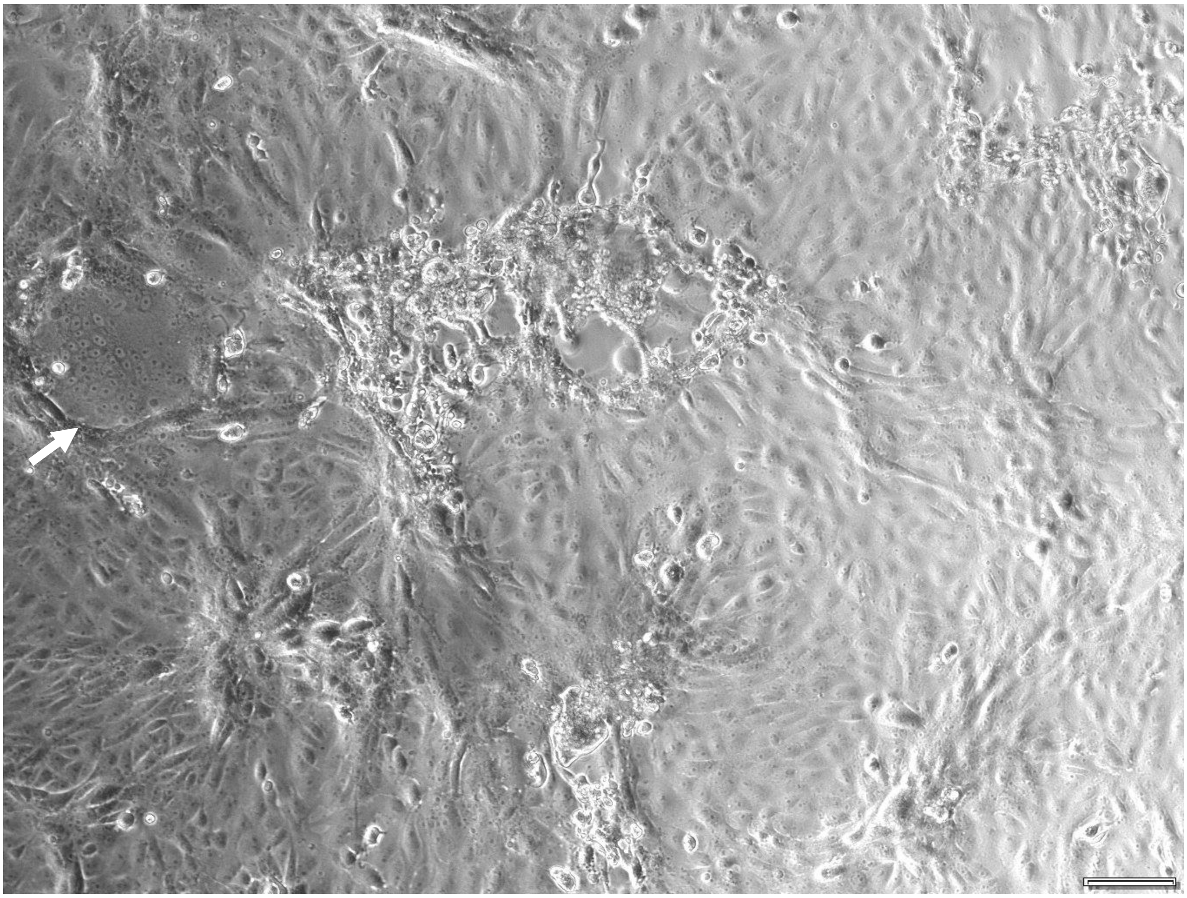

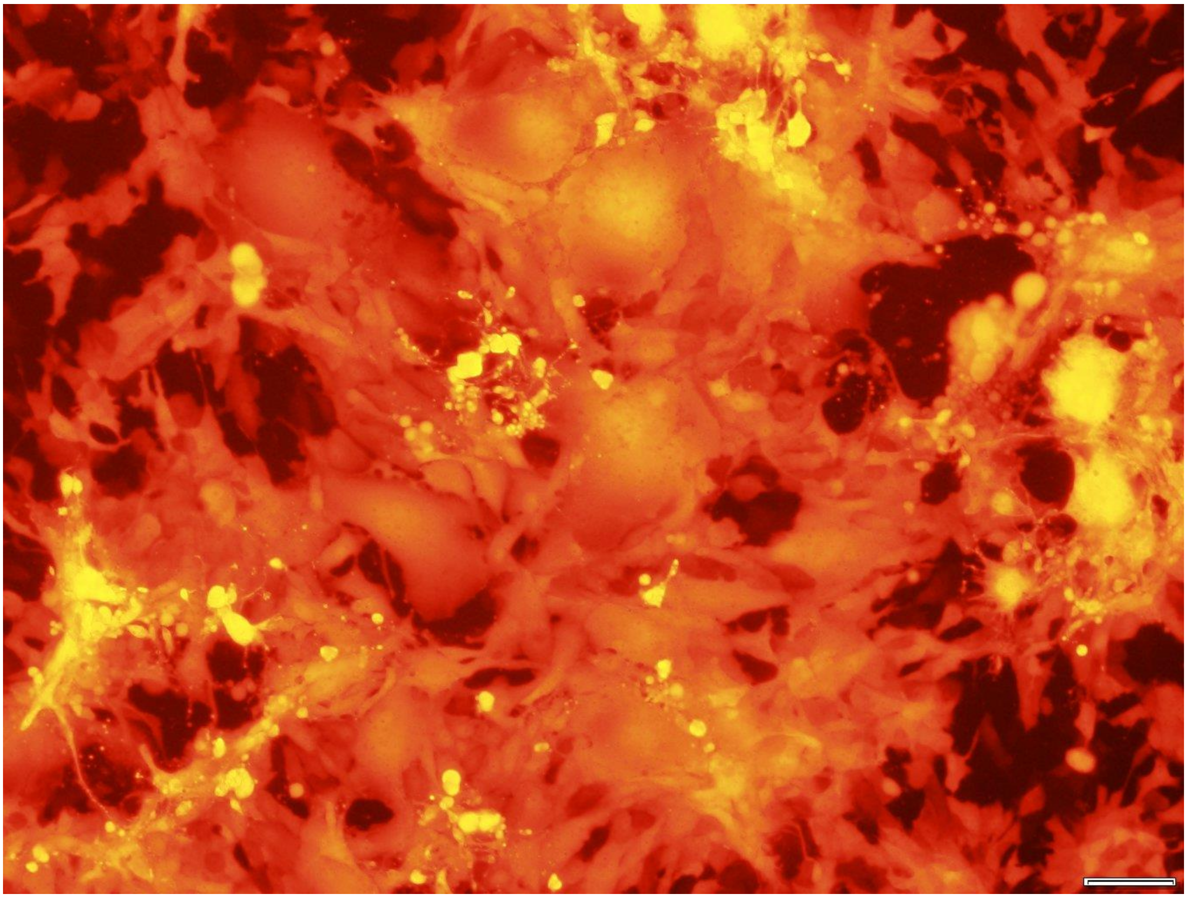

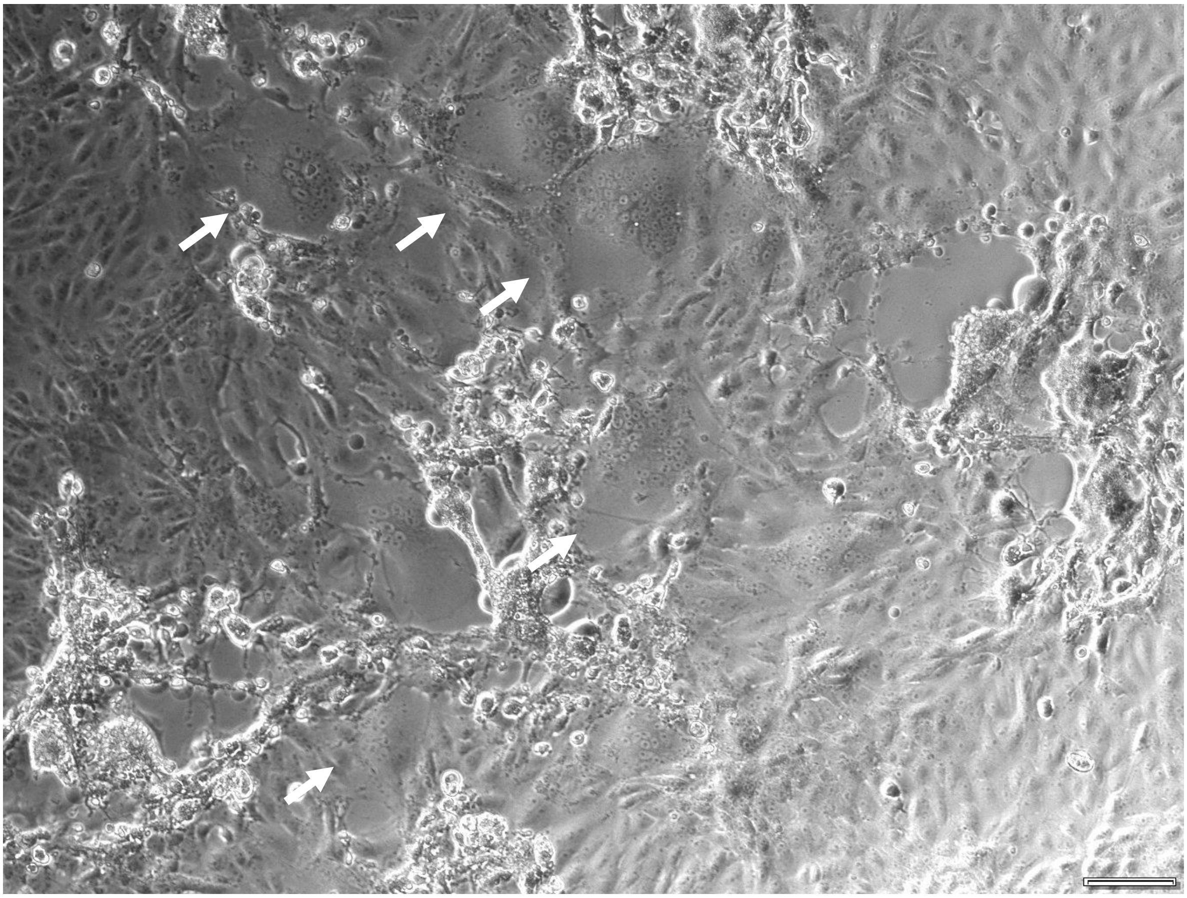

Supplementary Figure 1. Raw data related to Figure 1 and higher resolution images for data presented in Figure 1. (A) Normalized reads for each of the three repeats – shown in Fig. 1A. (B) FACS reads for each of the three repeats shown in Fig. 1B as well as a summary table for the data shown in Fig. 1B. Higher resolution images of DMSO treated Vero E6 cells showing syncytia (White arrow head) at (C) day 4 and (D) at day 5 of the virus spread. White arrow head marks syncytia.

Supplementary figure 2

A.

Concentration uM	% of no Drug		
0.78125	114.2099	115.2338	107.6053
1.5625	108.0839	103.6846	108.9299
3.125	104.2638	105.5972	110.3894
6.25	105.3189	108.0581	114.4753
12.5	96.95333	95.61943	107.2876
25	16.37594	9.014835	51.29215
50	0.147323	0.170218	0.212706

B.

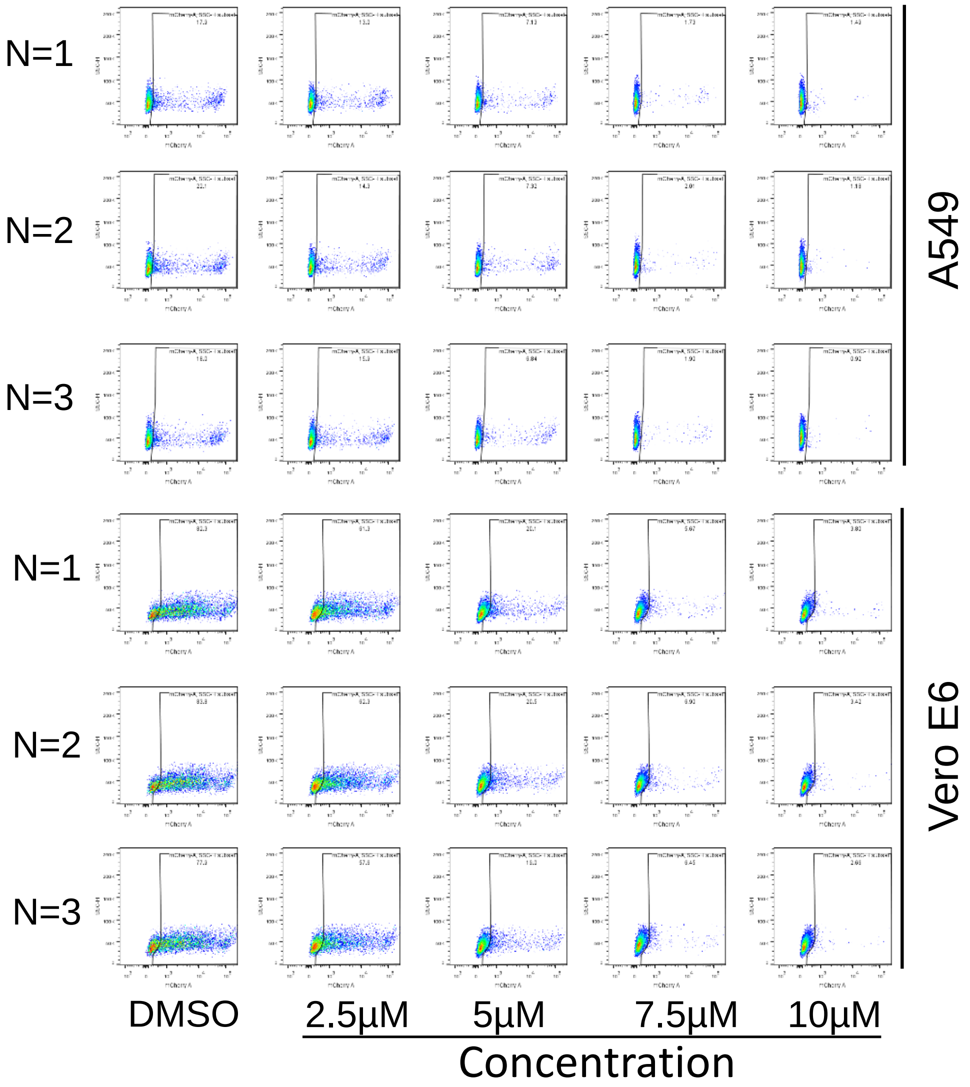

Concentration uM	A549			Vero E6		
DMSO	100	100	100	100	100	100
2.5	72.6257	64.70588	88.33333	74.4836	74.34368	73.94095
5	39.8324	33.12217	38	24.42284	24.46301	24.39024
7.5	9.664804	9.095023	10.55556	6.889429	8.23389	8.279846
10	8.324022	5.339367	5.111111	4.617254	4.081146	3.414634

Supplementary Figure 2. Raw data related to Figure 2. (A) Normalized reads for each of the three repeats – shown in Fig. 2A. (B) FACS reads for each of the three repeats shown in Fig. 2D as well as a summary table for the data shown in Fig. 2D.

Supplementary figure 3

A.

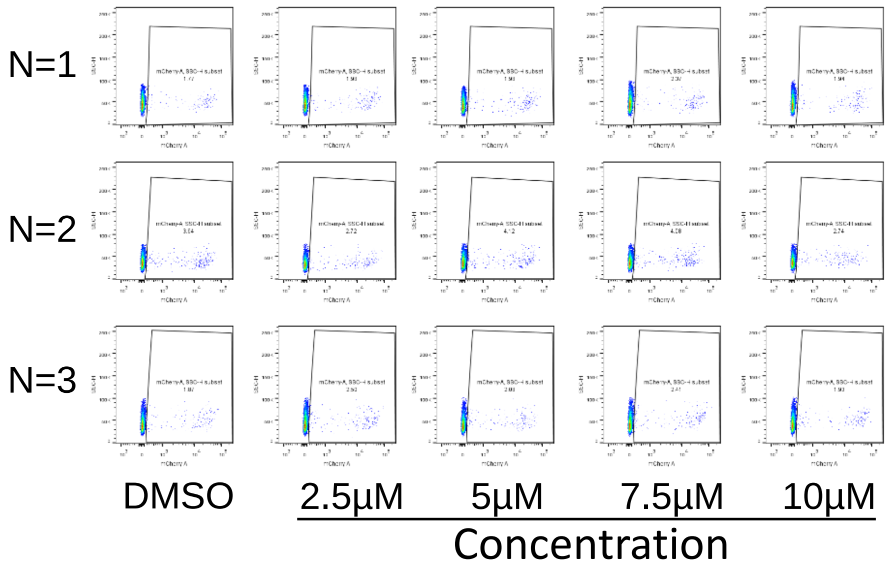

Concentration uM	Mean % to DMSO	SEM
DMSO	100	0
2.5	106.7598	17.21189
5	112.0937	0.576417
7.5	124.9544	6.594528
10	96.02927	10.54025

B.

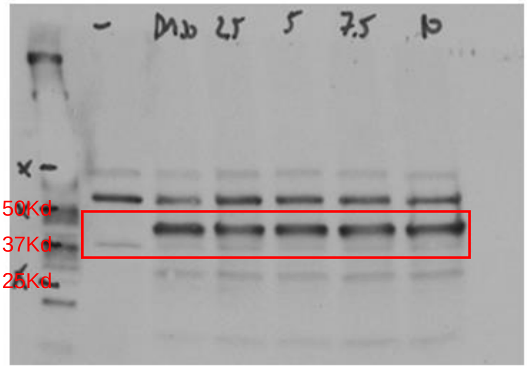

Cell Lysate anti-hRSV N

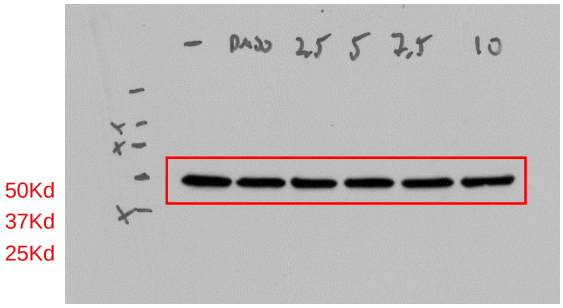

Cell Lysate anti-GAPDH

C.

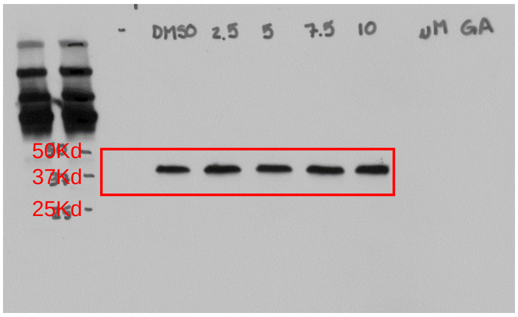

Viral Pellet anti-hRSV N

D.

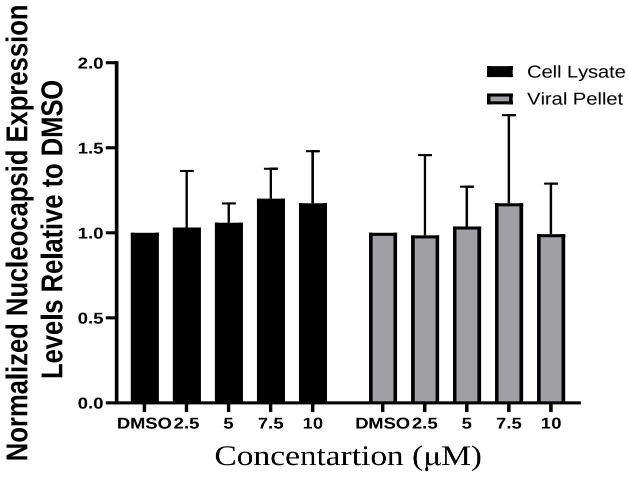

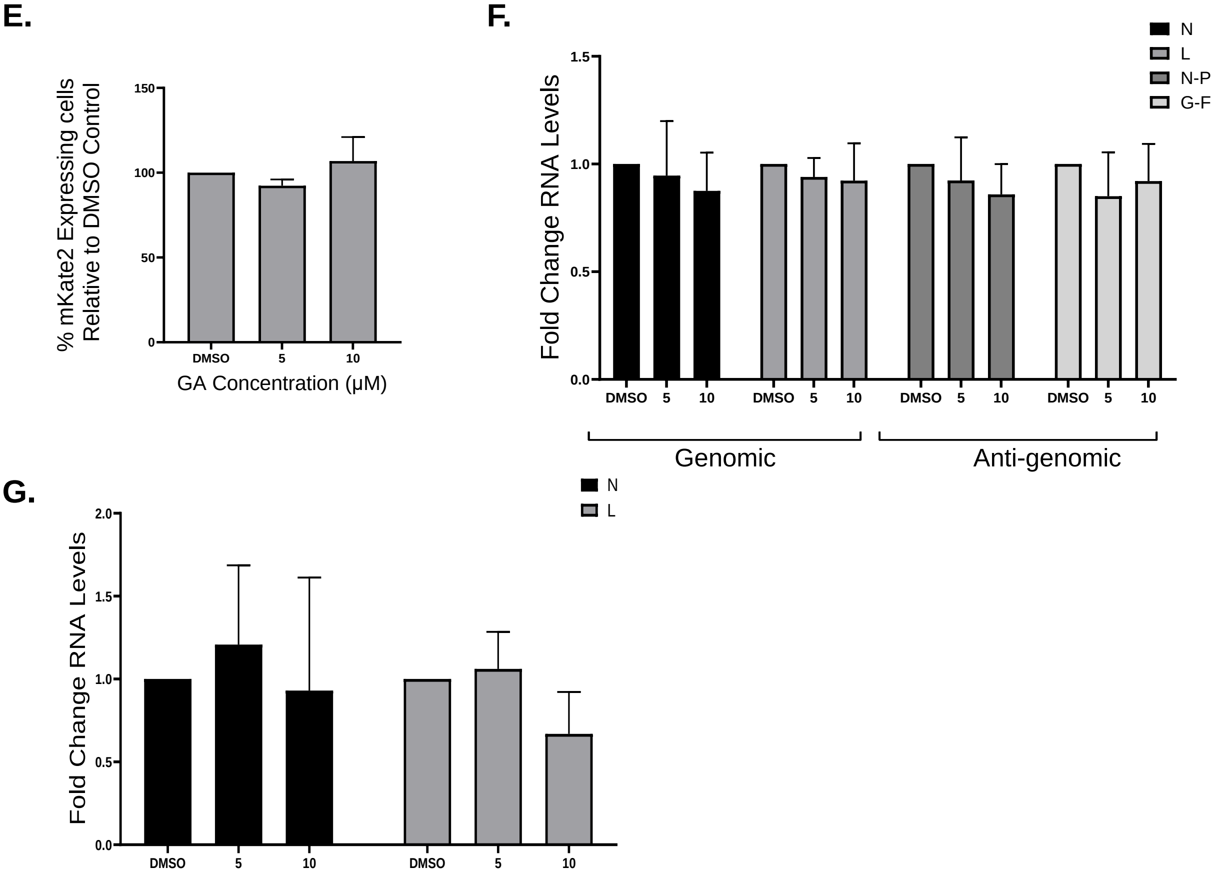

Supplementary Figure 3. Raw data related to Figure 3 and analysis of Respiratory syncytial virus replication, assembly and release post 48 h of Ginkgolic acid treatment. (A) FACS reads for each of the three repeats shown in Fig. 3B as well as a summary table for the data shown in Fig. 3B. (B) Uncropped WBs of data shown in Fig. 3C. (C) Uncropped WBs of data shown in Fig. 3E. (D) Densitometric analysis of data presented in Fig. 3C and E. shown are relative changes in N protein expression levels (measured using the anti-RSV N antibody) relative to DMSO treated cells, all normalized to GAPDH for each experiment. The data are mean ± SEM from three independent experiments (n = 3). (E-G) A549 cells were treated with DMSO or with the indicated GA concentrations for 24 h. Post treatment the cells were infected for 2 h with hRSV-mKate2 in the absence of the drug, after which fresh media containing the original drugs concentrations were used to replaced the infection media, and the cells were incubated in the presence of the drug for additional 24 h. (E) FACS analysis to quantify the number of mKate2 expressing cells after the full 48 h. The percent live cells scoring red-positive relative to DMSO control (set as 100) are plotted. The data are mean ± SEM from three independent experiments (n = 3). (F) Viral genomic and anti-genomic RNA levels form cell lysates were assessed using quantitative reverse transcription polymerase chain reaction (qRT-PCR). Two different primer sets were used for each of the RNA species - primers for N and L ORFs were used to measure genomic RNA levels, and primers for the regions spanning between N and P ORFs or F and M2 ORFs were used to measure the levels of the anti-genomic RNAs. The fold change of viral genomic or anti-genomic RNA levels normalized to microtubules mRNA levels in GA treated compared to DMSO treated are plotted. The data are mean ± SEM from three independent experiments (n = 3). (G) qRT-PCR of virion pellets using two primer sets (amplifying in N and L ORFs) was used to measure the levels of genomic RNA in each sample. The fold change of genomic RNA levels in GA treated compared to DMSO treated are plotted. The data are mean ± SEM from three independent experiments (n = 3).

Supplementary figure 4

A.

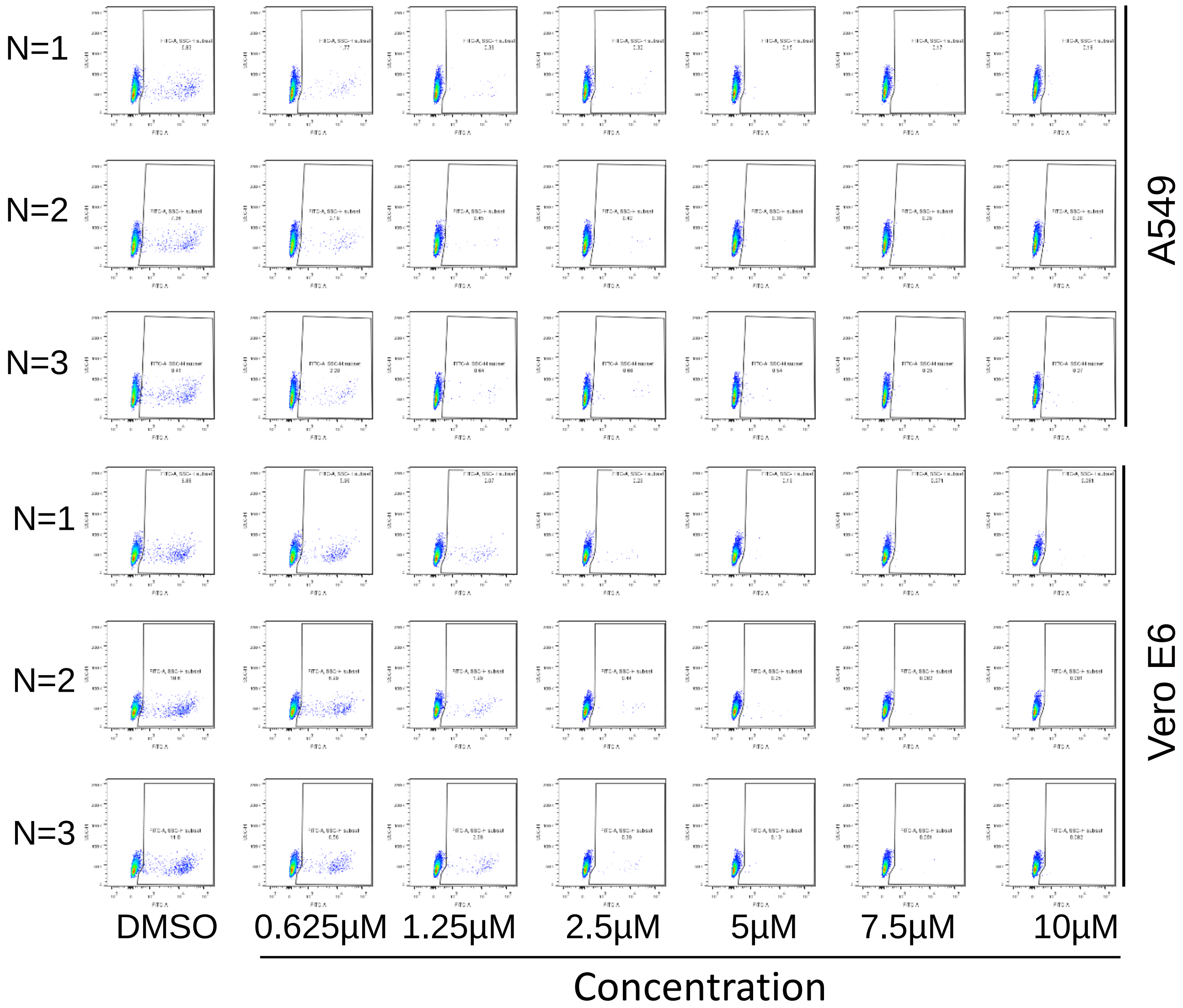

Concentration uM		A549			Vero E6	
DMSO	100	100	100	100	100	100
0.625	27.43733	29.75543	26.15933	67.11712	69.4831	59.63636
1.25	7.938719	6.11413	7.609988	23.31081	19.78131	24.45455
2.5	7.24234	5.706522	7.8478	3.265766	4.373757	3.545455
5	3.481894	4.076087	6.420927	2.13964	2.485089	1.181818
7.5	5.013928	3.940217	2.972652	0.79955	0.815109	0.463636
10	4.5961	2.717391	3.210464	0.912162	0.805169	0.745455

B.

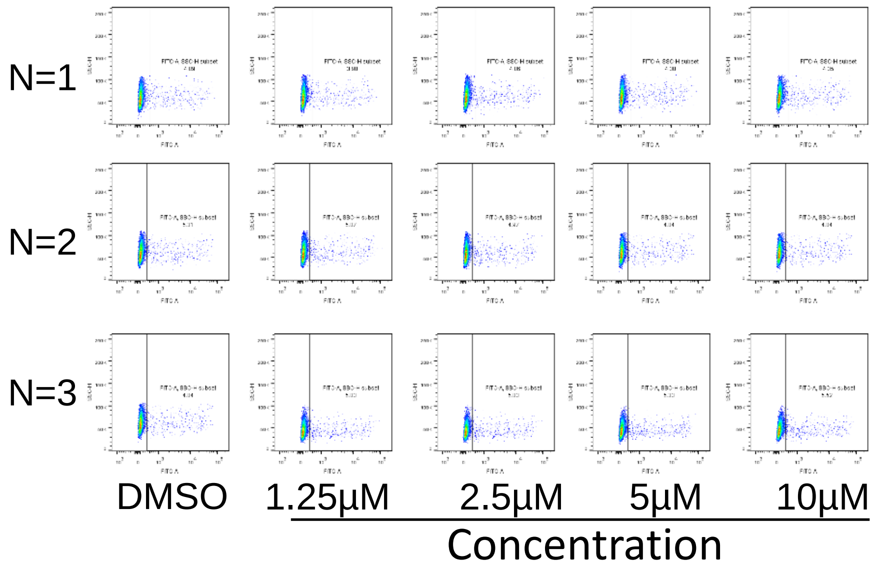

Concentration uM	Mean % to DMSO	SEM
DMSO	100	0
1.25	98.25346	2.836314
2.5	101.2024	5.043616
5	97.11377	10.61364
10	95.20703	5.981946

C.

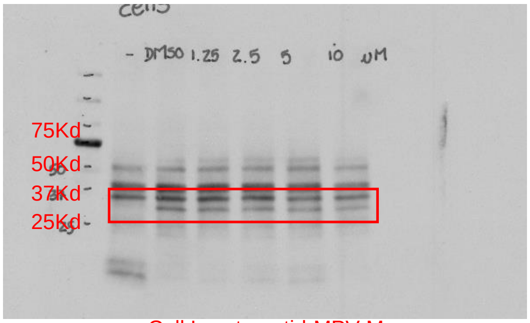

Cell Lysate anti-hMPV M

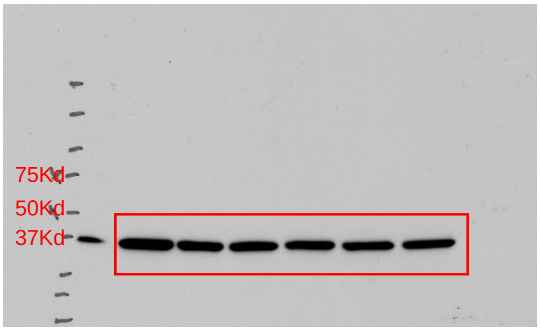

Cell Lysate anti-GAPDH

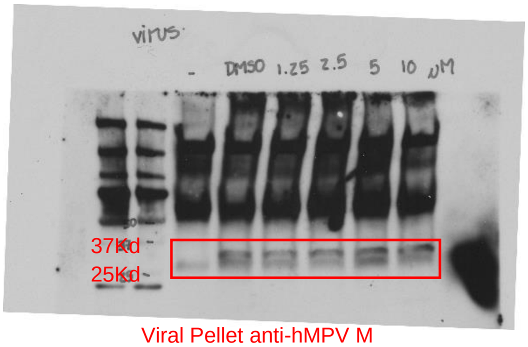

Viral Pellet anti-hMPV M

D.

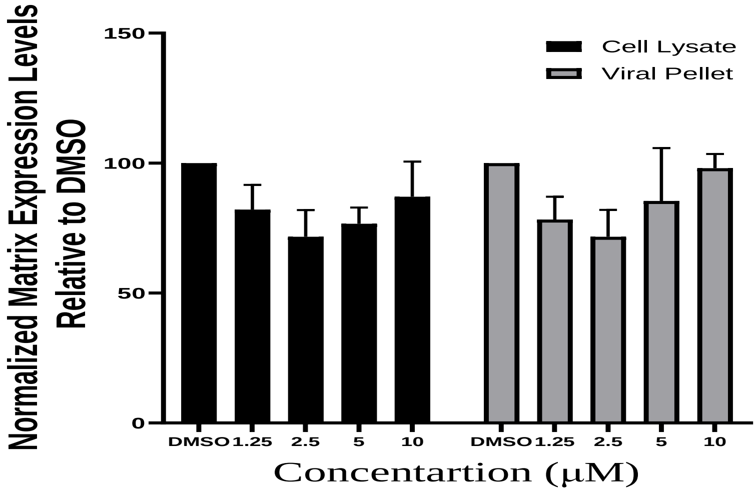

E.

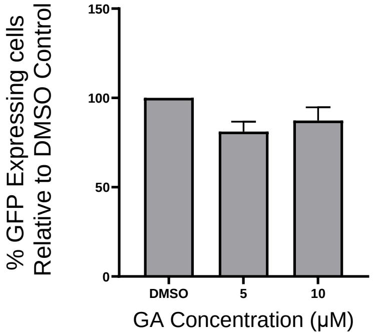

F.

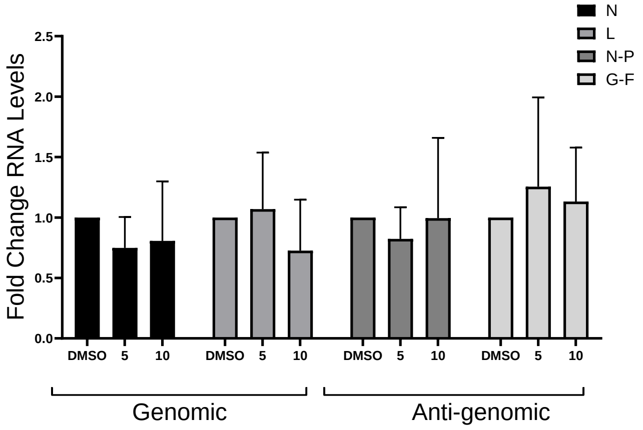

G.

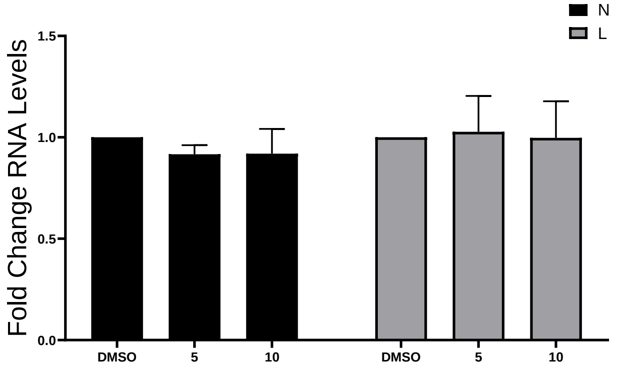

Supplementary Figure 4. Raw data related to Figure 4 and analysis of human Metapneumovirus replication, assembly and release post 48 h of Ginkgolic acid treatment. (A) FACS reads for each of the three repeats shown in Fig. 4C as well as a summary table for the data shown in Fig. 4C. (B) FACS reads for each of the three repeats of shown in Fig. 4D as well as a summary table for the data shown in Fig. 4D. (C) Uncropped WBs of data shown in Fig. 4E. (D) Densitometric analysis of data presented in Fig. 4E. shown are relative changes in M protein expression levels (measured using the anti-hMPV M antibody) relative to DMSO treated cells, all normalized to GAPDH for each experiment. The data are mean ± SEM from three independent experiments (n = 3). (E-G) A549 cells were treated with DMSO or with the indicated GA concentrations for 24 h. post treatment the cells were infected for 2 h with hMPV-GFP in the absence of the drug after which fresh media containing the original drugs concentrations were used to replaced the infection media, and the cells were incubated in the presence of the drug for additional 24 h. (E) FACS analysis to quantify the number of GFP expressing cells after the full 48 h. The percent live cells scoring green-positive relative to DMSO control (set as 100) are plotted. The data are mean ± SEM from three independent experiments (n = 3). (F) Viral genomic and anti-genomic RNA levels form cell lysates were assessed using quantitative reverse transcription polymerase chain reaction (qRT-PCR). Two different primer sets were used for each of the RNA species - primers for N and L ORFs were used to measure genomic RNA levels, and primers for the regions spanning between N and P ORFs or F and M2 ORFs were used to measure the levels of the anti-genomic RNAs. The fold change of viral genomic or anti-genomic RNA levels normalized to microtubules mRNA levels in GA treated compared to DMSO treated are plotted. The data are mean ± SEM from three independent experiments (n = 3). (G) qRT-PCR of virion pellets using two primer sets (amplifying in N and L ORFs) was used to measure the levels of genomic RNA in each sample. The fold change of genomic RNA levels in GA treated compared to DMSO treated are plotted. The data are mean ± SEM from three independent experiments (n = 3).

Primer name	Sequence 5' -- > 3'	Usage
**h_N_qPCR_F**	CTGTAAGCCCTAACGAAGAGAAG	Genomic hMPV N forward
**h_N_qPCR_R**	TACAGAGGGAGAGTACCAAACA	Genomic hMPV N reverse
**h_L_qPCR_F**	AGAAGAGCTGATTCGGAGATAGA	Genomic hMPV L forward
**h_L_qPCR_R**	GCAGTAGGATCAGAACGACAAG	Genomic hMPV L reverse
**h_ag_NP_qPCR_F**	ACATGAGTGGTGACAATCAAGA	Anti-genome hMPV N to P forward
**h_ag_NP_qPCR_R**	TCTGGAAAGCTTCGGCTATTT	Anti-genome hMPV N to P reverse
**h_ag_FM2_qPCR_**	CGGCGGTTTCATACCACATA	Anti-genome hMPV F to M2 forward
**h_ag_FM2_qPCR_R**	ATCACTCCCTCTGTTGCATTT	Anti-genome hMPV F to M2 reverse
**r_N_qPCR_F**	TGGCTATGTCCTTGGGTAGTA	Genomic hRSV N forward
**r_N_qPCR_R**	CTGGTCTTACAGCCGTGATTAG	Genomic hRSV N reverse
**r_L_qPCR_F**	GCCTATGCCTGCATACTCTTTA	Genomic hRSV L forward
**r_L_qPCR_R**	TCAGACTCATGGAAGGTCAAAC	Genomic hRSV L reverse
**r_ag_NP_qPCR_F**	CAGCAGAAGAACTAGAGGCTATC	Anti-genome hRSV N to P forward
**r_ag_NP_qPCR_R**	CCATGGAATTCAGGAGCAAAC	Anti-genome hRSV N to P reverse
**r_ag_GF_F**	ACCACGCCAGTAGTTACTTAAA	Anti-genome hRSV F to M2 forward
**r_ag_GF_R**	TGCATTTGCTTTGAGGATTGG	Anti-genome hRSV F to M2 reverse
**MT_qPCR_F**	ACCTTAACCGCCTTATTAGCCA	Microtubules forward
**MT_qPCR_R**	ACATTCAGGGCTCCATCAAATC	Microtubules reverse

Table. 1 primers used in this study
